# Evolution and functional diversification of R2R3-MYB transcription factors in plants

**DOI:** 10.1093/hr/uhac058

**Published:** 2022-03-08

**Authors:** Yun Wu, Jing Wen, Yiping Xia, Liangsheng Zhang, Hai Du

**Affiliations:** 1 Department of Landscape Architecture, School of Civil Engineering and Architecture, Zhejiang Sci-Tech University, Hangzhou, 310018, China; 2 College of Agronomy and Biotechnology, Southwest University, Chongqing, 400716, China; 3 Genomics and Genetic Engineering Laboratory of Ornamental Plants, College of Agriculture and Biotechnology, Zhejiang University, Hangzhou, 310058, China

## Abstract

R2R3-MYB genes (*R2R3-MYBs*) form one of the largest transcription factor gene families in the plant kingdom, with substantial structural and functional diversity. However, the evolutionary processes leading to this amazing functional diversity have not yet been clearly established. Recently developed genomic and classical molecular technologies have provided detailed insights into the evolutionary relationships and functions of plant *R2R3-MYBs*. Here, we review recent genome-level and functional analyses of plant *R2R3-MYBs*, with an emphasis on their evolution and functional diversification. In land plants, this gene family underwent a large expansion by whole genome duplications and small-scale duplications. Along with this population explosion, a series of functionally conserved or lineage-specific subfamilies/groups arose with roles in three major plant-specific biological processes: development and cell differentiation, specialized metabolism, and biotic and abiotic stresses. The rapid expansion and functional diversification of plant *R2R3-MYBs* are highly consistent with the increasing complexity of angiosperms. In particular, recently derived *R2R3-MYBs* with three highly homologous intron patterns (a, b, and c) are disproportionately related to specialized metabolism and have become the predominant subfamilies in land plant genomes. The evolution of plant *R2R3-MYBs* is an active area of research, and further studies are expected to improve our understanding of the evolution and functional diversification of this gene family.

## Introduction

The V-myb avian myeloblastosis viral oncogene homolog (MYB) genes encode a group of pan-eukaryotic transcription factors (TFs) characterized by a highly conserved N-terminal DNA-binding domain repeat (R) plus a variable C-terminal regulatory region [1–6]. MYB genes constitute the second largest TF superfamily in flowering plants [7], accounting for ~13% of the 1500 TFs in the model plant *Arabidopsis thaliana* [8]. Conversely, only a few MYB genes are present in unikonts [9, 10]; for example, only three have been reported in the *Homo sapiens* genome, the human c-MYB proto-oncogene product (c-MYB) and two related vertebrate MYB factors [11, 12]. Each MYB repeat contains approximately 50 amino acid residues forming three α-helices and special DNA motifs, including MYB binding site I (MBSI), MBSII, or MBSIIG [12–15], with some exceptions [16]. According to sequence similarity, MYB domain repeats are classified as R1, R2, or R3 type [5, 17]. Typically, plant MYB superfamily members include 1–4 imperfect MYB repeat(s) and are therefore subdivided into four families: 1R-MYB (consisting of one or two separated repeats), 2R-MYB (R2R3-MYB, consisting of two adjacent repeats), 3R-MYB (consisting of three adjacent repeats), and 4R-MYB (consisting of four adjacent repeats) [5]. The R2R3-MYB family has expanded substantially in the plant lineage and is the predominant family [6, 18, 19].

Since the first plant R2R3-MYB gene, *COLORED1 (C1)*, was identified in *Zea mays* and was demonstrated to be essential for anthocyanin biosynthesis in aleurone tissues [20], a tremendous number of R2R3-MYB genes (R2R3-MYBs) have been identified. In the past decade, based on substantial genome sequence data, the whole R2R3-MYB gene family has been comprehensively identified in numerous plant genomes (Supplemental Table 1), with counts ranging from dozens to hundreds, e.g. 126 genes in *A. thaliana* [9], 192 in *Populus* [21], 244 in soybean [22], and 406 in *Gossypium hirsutum* [23]. A comparative phylogenetic analysis of R2R3-MYBs revealed considerable diversification and conservation of this gene family in plants [10]. Concomitantly, the functions and characteristics of R2R3-MYB proteins have been studied extensively (Supplemental Table 2), including their roles in various plant-specific processes, such as responses to ambient stimuli [24–29], specialized metabolism [5, 20, 30–34], development [35–38], and cell differentiation [39–41]. Given their important roles in plants, the separate biological processes regulated by R2R3-MYBs have recently been reviewed in detail elsewhere [42–47].

A number of studies have shown that this gene family underwent a large expansion with functional diversification in land plants [6, 9, 10, 18, 19, 21, 49]. This may have contributed to the origin and diversity of this kind of plant, as it conferred on them the ability to adapt from aquatic to terrestrial environments during evolution [50]. To capture the most significant developments in this area, this review focuses on (a) the identification and classification of the R2R3-MYB gene family in plants, (b) the characterization of conserved and diverse functions in different species and biological processes, and (c) the use of comparative genomics to clarify the expansion and functional diversification of the R2R3-MYB family. Finally, we discuss new research directions to improve our understanding of R2R3-MYB evolution and functions.

## Genome-wide characteristics and evolution of the R2R3-MYB gene family in plants

### Identification and classification of R2R3-MYBs

**Figure 1:**
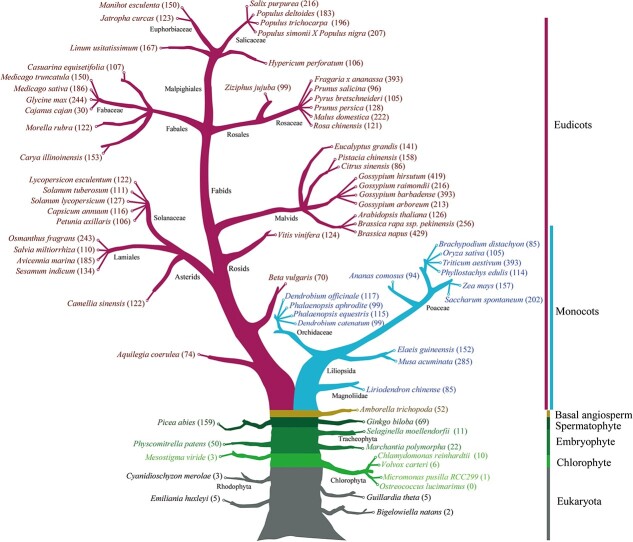
Phylogenetic relationships of 75 plant species in which the *R2R3-MYB* gene family has been comprehensively analyzed. Phylogenetic relationships among these species were obtained from the NCBI taxonomy database (https://www.ncbi.nlm.nih.gov/taxonomy). In total, 10 270 sequences of 75 plant species from Rhodophyta to angiosperms have been reported. Numbers in round brackets indicate the number of *R2R3-MYB* family members in each species. Detailed information, including corresponding references for each species, is provided in Supplemental Table 1. The end date for these statistics is November 2021.

Since its initial identification in 1982 [1], the R2R3-MYB family has been an important area of research. In the past 20 years, an increasing number of sequenced plant genomes have enabled integrative genome-wide overviews of this gene family in the plant kingdom. To date, the R2R3-MYB gene family has been evaluated in about 74 plant species, ranging from aquatic plants (such as *Mesostigma viride* [51]) to angiosperms (e.g. *A. thaliana* [9], *Z. mays* [49], *Populus trichocarpa* [21], *Gossypium* spp. [23], and *Solanum tuberosum* [52]) (Fig. 1 and Supplemental Table 1), and a pipeline for the automatic identification of MYBs has been created in order to make genome- or transcriptome-wide investigations more consistent [53]. These studies have revealed that *R2R3-MYBs* are widely distributed in the plant kingdom, with a sharp increase in number from aquatic to angiosperm plants, forming one of the largest and most diverse TF gene families (ranging from one to hundreds of members) in land plants, especially angiosperms (Fig. 1).

The R2R3-MYB gene family clearly underwent functional diversification to form different subfamilies/groups during evolution. Accordingly, there are many classification schemes for plant R2R3-MYBs, and the differences primarily reflect the existence of lineage- or species-specific “orphan” genes [54, 55], differences in sampling coverage, and phylogenetic analysis methods [6]. On the whole, three landmark classification systems have been developed and improved. First, the canonical classification and nomenclature of this gene family were initially established in *A. thaliana* [9] and included 25 groups based on the MYB domain and the amino acid motifs in the C-terminal regions [5]. Thereafter, many studies have applied this system with varying degrees of modification [22, 49, 56–58]. Notably, in *A. thaliana* [9], around 30 of the 126 (~23.81%) *R2R3-MYBs* were not assigned to any subfamily/subgroup in this classification owing to the lack of different representative species and the limited gene number (generally only one per species), resulting in scattered studies with relatively small sample sizes. Second, we performed a systematic comparative analysis of 50 major eukaryotic lineages with a total of 1548 *R2R3-MYBs* [10]. As expected, we not only confirmed the 25 well-defined subfamilies of the R2R3-MYB gene family in *A. thaliana* [9] but also defined many new species- or lineage-specific subfamilies in major eukaryotic lineages that had previously been neglected [21]. Ultimately, we classified the gene family into as many as 73 subfamilies supported by highly conserved gene structures and motif compositions [10]. The validity of this classification was further supported by our recent study of Brassicaceae [18]. In addition, a more systematic classification with a high resolution based on 87 species and 4312 sequences has recently been released, covering the major lineages of Archaeplastida. In that study, ten clades were designated as land plant R2R3-MYB subfamilies by applying a progressive phylogenetic analysis strategy aimed at eliminating the imbalance in resolution and species sampling [6]. We speculated that there may be more subfamilies specific to individual species or lineages, as there are still many “orphan” genes in the phylogenetic analyses. Third, the genome sequences for *Marchantia polymorpha* [59] and *M. viride* [51] have enabled the development of a macroscopic classification regime, resulting in three clades of *R2R3-MYBs* that correspond to their biological functions. Each clade is correlated with different functions associated with essential developmental processes, from the basic life cycle to specialized metabolism and organismal complexity, corresponding to a very recent classification in 2020 [60]. In that study, genes encoding R2R3-MYBs from Rhodophytes, Glaucophytes, Chromista, Chlorophytes, Charophytes, and Embryophytes were divided into three major clades (I, II, and III) (named subgroups in the original paper). Subgroup I is the most ancient group and includes members from all plant lineages, whereas subgroup III has become predominant in land plants [60]. The former two classification schemes share overlaps and are based on domain conservation, whereas this final classification system provides a macroscopic perspective based on broad evolutionary patterns.

Notably, a uniform nomenclature within the classification of this gene family in plants is still lacking. Currently, there is almost no dispute at the family (R2R3-MYB) and superfamily (MYB) levels, except for a few studies that refer to *R2R3-MYBs* as a subfamily or subgroup [50, 61]. However, the nomenclature after R2R3-MYB is inconsistent, e.g. ~7% of studies refer to groups [62, 63], ~31% refer to subfamilies [10, 64], and ~54% refer to subgroups [23, 65], with clade or cluster used occasionally [66, 67]. Subfamilies and subgroups are the most common subsequent appellations after family. Given that subfamily is used after the family level in many other TF gene families, e.g. basic helix–loop–helix proteins (bHLHs) [68], MADS-Box [69], and GRAS [70], we propose an improved nomenclature system from superfamily (MYB) and family (R2R3-MYB) to subfamily. “Subgroup” used by Chang and colleagues should be revised to evolutionary clade/group [60]. To avoid confusion, in the following sections, we uniformly refer to the lower classifications within the gene family as subfamilies (abbreviated as S1, S2, etc.).

### Structural conservation of R2R3-MYBs

**Figure 2:**
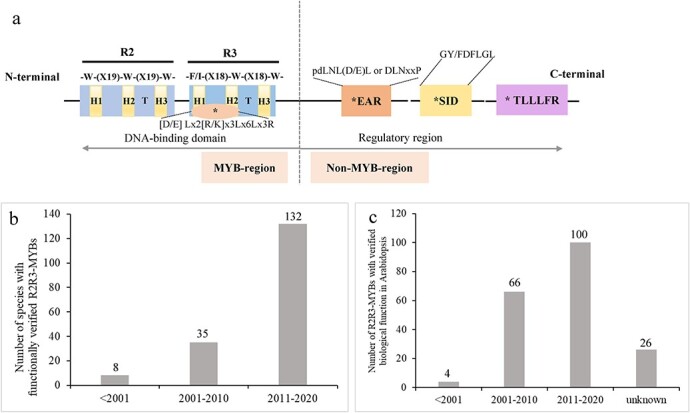
Domain structure and functional characterization status of R2R3-MYB transcription factors. a. The R2R3-MYB transcription factor is composed of a MYB region and a non-MYB region. The DNA-binding domain, also called the MYB region, contains conserved R2 and R3 repeats. In most activators and some repressors [43], there is a conserved bHLH-interacting motif ([D/E] Lx2[R/K]x3Lx6Lx3R) within the first two helixes of the R3 domain that enables interactions with bHLH proteins [48] to form the MYB-bHLH-WDR transcriptional complex. The protein sequences in the C-terminal region often show divergence, with one or a few typical repressor motif(s) such as the EAR motif (ERF-associated amphiphilic repression), SID motif (Sensitive to ABA and Drought 2 protein interact motif), and TLLLFR. H, Helix; T, turn; W, tryptophan; X, amino acid. ^*^ indicates that the motif is not included in all R2R3-MYBs, but only in some. b. Number of species for which the functions of one or more *R2R3-MYB* genes were identified as of 2020. In the last decade (2011–2020), there was explosive growth in the breadth of taxa for which *R2R3-MYB* data were available. It is noteworthy that most of the increase in species number is ascribed to the vast number of orthologous MYB genes characterized in horticultural plants that have similar functions, especially in phenylpropanoid biosynthesis. c. Number of *Arabidopsis* R2R3-MYBs for which biological role(s) have been identified. In *Arabidopsis*, all the increase is due to paralogs with new functions. When a gene had different functions published in different years, we sorted it into the year of the first publication. The dark grey bar represents the number of R2R3-MYBs with unknown (i.e. not experimentally verified) functions.

The R2R3-MYB protein consists of two major functional parts: a DNA-binding domain (MYB domain) located at the N terminus and a regulatory region (non-MYB region) located at the C terminus. The MYB domain is a signature, highly conserved feature in the gene family, whereas the non-MYB regions have diverged among plant species (Fig. 2a). In addition, the intron patterns in the MYB domains of the *R2R3-MYBs* are highly conserved within each subfamily across land plants [6, 9, 10, 22, 49]. In most cases, the MYB domain has one or two conserved intron insertion sites, with rare intron-less and multi-intron genes [6, 10]. Although the number of intron patterns differs slightly among taxonomic groups, there is clearly high structural conservation of MYB domains across land plants. In our previous study, we identified 12 highly conserved intron patterns across the plant kingdom [10] that may have arisen early in the transition from aquatic to terrestrial plants based on conserved intron numbers, insertion positions, and intron phases (Supplemental Fig. 1). The majority (~70%) of the tested *R2R3-MYBs* share three types (patterns a, b, and c) of the 12 intron patterns (patterns a to l) [10], suggesting biased expansion during evolution. A recent study has shown that some charophyte *R2R3-MYBs* exhibit intron–exon structures identical to those of their corresponding land homologs in the same subfamily, whereas others show clear differences, indicating a difference among ancestral genes [6].

R2R3-MYB proteins can act as transcriptional activators as well as repressors [42]. Most are activators that bind to cis-elements in gene promoters to recruit transcriptional machinery and stimulate gene expression [43]. For instance, many R2R3-MYBs have a bHLH-interacting motif “(D/E)Lx2(R/K)x3Lx6Lx3R” in the R3 MYB domain (Fig. 2a), enabling physical interactions with the bHLH protein to form the MYB-bHLH-WDR (MBW) transcriptional complex [48]. Notably, although the conservation of the non-MYB region is much lower than that of the MYB domain, the C-terminal regions of closely related members within each subfamily or close subfamilies in an evolutionary clade generally share short conserved protein motifs (some called short linear motifs, SLiMs), such as EAR (ethylene-responsive element binding, “pdLNL(D/E)L” or “DLNxxP” in S4) [71], “(R/K)PRPRx(F/L)” in S6, SID (sensitive to ABA and Drought 2 protein interact motif), and TLLLFR [72] (Fig. 2a), indicating their common origin, functional similarities, and structural conservation [9]. Recently, Millard et al. [54] reported that the interactions between MYB TFs from S12 and bHLH were mediated by one of the conserved motifs ([L/F]LN[K/R]VA) located in the center of the non-MYB region. Two novel SLiMs with roles in protein–protein interaction were uncovered by Rodrigues et al. [73] in the disordered C-terminal region. For more detailed information, see the recent reviews by LaFountain and Yuan [47] and Chen et al. [35]. MYB activators and repressors often operate in a hierarchy [74]. Furthermore, many subfamily- and/or branch-specific motifs have been detected within R2R3-MYBs. For instance, based on a large number of species, up to 102 highly conserved motifs have been identified in non-MYB regions [10]. Interestingly, non-MYB regions across the entire *A. thaliana* R2R3-MYB gene family contain extensive intrinsically disordered regions (IDRs) with special post-translational modification sites, binding sites for physical interactions, and activation domains. These features may explain the substantial functional diversity and the structure–function relationships in plant R2R3-MYBs [54]. However, owing to the high divergence in non-MYB regions, a systematic analysis of motif(s) and/or IDRs covering broad and representative plant lineages is still lacking. Moreover, further experimental evidence is needed, including for IDRs.

### Functional conservation and diversification of *R2R3-MYBs* in land plants

The biological functions of *R2R3-MYBs* have been verified in an increasing number of non-model crops with high economic value, especially in the last decade. More than 500 *R2R3-MYBs* have been functionally characterized in around 130 plant species (Fig. 2b and Supplemental Table 2), and their roles in many biological processes have been reported [5, 27–29, 32–34, 38, 41, 75–77]. Notably, research progress related to R2R3-MYB gene function is based mainly on studies of the model plant *A. thaliana*; approximately 80% of its 126 R2R3-MYBs have been experimentally characterized (Fig. 2c). Overall, the functions of *R2R3-MYBs* can be classified into three major processes: development and cell differentiation, specialized metabolism (especially the phenylpropanoid biosynthesis pathway), and stress responses (biotic and abiotic stresses).

### R2R3-MYBs are required for developmental and cell differentiation processes

Plant *R2R3-MYBs* are involved in the regulation of many important aspects of plant growth and development (Supplemental Table 2), e.g. trichome initiation and branching [78, 79], cuticle development [80], and regulation of different organs, including the flower, seed [81, 82], shoot [41, 83, 84], and root [85, 86]. In general, genes from the S9 (e.g. *AtMYB16*), S15 (e.g. *AtMYB66*), S18 (e.g. *AtMYB33*), S25 (e.g. *AtMYB64*), and S27 (e.g. *AtMYB91*) subfamilies [9] are exclusively associated with the above-mentioned biological functions (Supplemental Table 2). For instance, genes in S9 are involved in cuticle formation and trichome branching in *A. thaliana* (*AtMYB16* and *AtMYB106*) [80] and *M. polymorpha* (*MpSBG9*) [87], conical epidermal cell outgrowth in *Antirrhinum majus* (*AmMYBML1*) [88], photosynthesis and growth in *Betula platyphylla* (*BpMYB106*) [89], and fiber development in cotton (*GhMML3*) [90]. The genes in S18 mainly regulate the development of flower organs such as stamens (*AtMYB33*, *AtMYB65*, and *BcMF28*) [91, 92] and anthers (*OsGAMYB*) [93], as well as pollen tube–synergid interactions (*AtMYB97*, *AtMYB101*, and *AtMYB120*) [94]. Interestingly, the above processes are usually mediated by hormones and/or hormone signaling, e.g. JA and GA. *R2R3-MYBs* (e.g. members of S25) are also involved in plant reproductive growth [95] (Supplemental Table 2). More recently, *MpFGMYB*, a member of S25, was identified as a critical factor in sexual dimorphism determination in *M. polymorpha* [96], and similar results regarding spore and sexual organ development were obtained in *Selaginella moellendorffii* (*SmGAMB*) and *Physcomitrella patens* (*PpGAMYB1* and *PpGAMYB2*) [97]. Members of S27 contribute to the regulation of vegetative organ formation, including leaf patterning and development (*NbPHAN* and *PHANTASTICA*) [98, 99], stomatal cell differentiation and formation [16], and axillary meristem formation (*ZmRS2* and *SlPHAN*) [100–102] (Supplemental Table 2). S14 also functions in meristem initiation as well as root formation (*CRY1*) [103, 104] (Supplemental Table 2). A good example in this subfamily is the *MpGCAM1* gene, which precisely controls gemma cup formation in liverworts [105]. Another example of genes that regulate cell growth during gametophytic development are *MpPp1* and *MpPp2* in *P. patens* [106]. Furthermore, some subfamilies have species-specific functions. For instance, *AtMYB21*, *AtMYB24*, and *AtMYB57* in S19 have key roles in stamen development [107, 108]. However, their homologs in almost all other species studied, including *Freesia* hybrids [109, 110], *Gentian tutea* [111], *Ficus carica* [112], *Malus domestica* [113], and *Petunia hybrida* [114], regulate specialized metabolism (Supplemental Table 2). Further research should focus on S16 [115], S24 [116], S28 [117], S31 [118], S33 [119], S36 [120], and S37 [121, 122], for which experimental data are rather limited (Supplemental Table 2).

In summary, *R2R3-MYBs* are a class of important TFs necessary for the basic life cycle of plants. Therefore, there is a need for more extensive field studies of *R2R3-MYBs* in the future to determine their functions in basic developmental processes.

### R2R3-MYBs respond to various environmental stresses

Over the past few decades, *R2R3-MYBs* have emerged as key regulators of responses to diverse abiotic stresses, such as drought, temperature, salinity, and phosphate starvation [28, 123–127] (Supplemental Table 2); see previous reviews [75, 128, 129]. Representative subfamilies are S1, S2, S11, S17, S20, S22, and S38. In *A. thaliana*, members of S1 (*AtMYB30*, *AtMYB60*, and *AtMYB96*) are involved in drought, heat, salt, and excess-light stresses [126, 130–137]; members of S2 (*AtMYB13*, *AtMYB14*, and *AtMYB15*) act as positive regulators of drought, wounding, and freezing tolerance [138–141]; *AtMYB41* and *AtMYB74* in S11 are involved in osmotic and/or salt stress [142, 143]; members of S20 (*AtMYB2*, *AtMYB62*, and *AtMYB108*) are responsive to a wide range of environmental stresses [144–146]; similar roles have been observed for S22 members (*AtMYB44*, *AtMYB73*, and *AtMYB77*) [26, 147, 148]. *AtMYB48* in S38 is involved in potassium stress possibly via alternative splicing, which modulates DNA-binding motifs [149]. Some genes from S3 [150], S4 [125], S8 [151], and S23 [152] also show stress-induced functionality in *A. thaliana*. In addition to the *R2R3-MYBs* in *A. thaliana*, around 70 genes identified in 26 species have also been found to participate in distinct abiotic stresses (Supplemental Table 2). As in *A. thaliana*, such proteins are abundant in S2 [153–155], S11 [156–158], S20 [159–161], and S22 [162–165]. However, distinct differences have been noted. Some groups have been confirmed only in *A. thaliana* (e.g. *AtMYB1* from S23a and *AtMYB72* from S3) [150, 152] or only in non-model species, such as S44, S4, S17, S28, S43, S7, S39, and S79 [123, 127, 166–178]. Taken together, these results suggest that *R2R3-MYBs* control responses to multiple abiotic stresses and act as major regulators. However, more research is needed to improve our understanding of the functional divergence and conservation of this gene family in plants. This understanding could provide a theoretical basis for the development of strategies to resolve the adverse effects of abiotic stresses, which are exacerbated by climate change.

Many R2R3-MYBs contribute to the biotic stress response, and at least 13 *R2R3-MYBs* have been reported to date (Supplemental Table 2). Interestingly, eight of these genes belong to S2. *OsMYB30* directly upregulates *OsPAL6* and *OsPAL8* to enhance resistance to the brown planthopper in rice [29]. The overexpression of *CmMYB15* in chrysanthemum confers resistance to aphids by regulating lignin biosynthesis [179]. *GmMYB29A2* regulates soybean resistance to *Phytophthora sojae* [180]. *SlHM1* and *SlMYB52* alter trichome density to confer spider mite tolerance in tomato via auxin signaling [118]. *VdMYB1* in *Vitis davidii* [181] and *TaRIM1* in wheat regulate pathogen defense [182]. Other examples include *AtMYB30* (S1) [183], *AtMYB96* (S1) [184], *AtMYB102* (S11) [185], and *AtMYB72* (S3) [186, 187] in *A. thaliana* and *OsJAmyb* (S39) in *Oryza sativa* [188].

In summary, *R2R3-MYBs* could improve plant responses to multiple abiotic and biotic stresses in agriculture and could be targets of advanced technologies, such as CRISPR technology, to improve crop resistance to environmental stimuli in order to maintain the food supply.

### Key roles of R2R3-MYBs in metabolite biosynthesis

**Figure 3:**
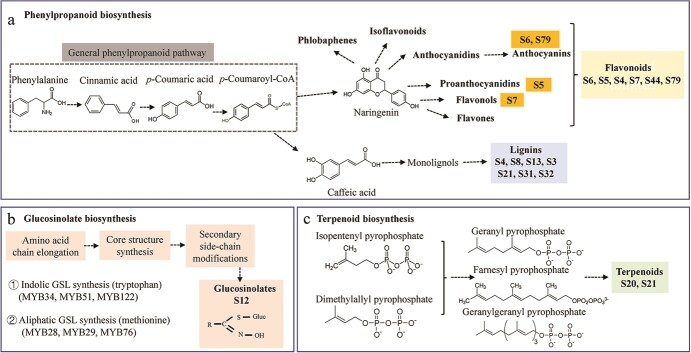
Major metabolite biosynthetic pathways regulated by R2R3-MYBs and simplified schemes are shown. a. The most intensively investigated regulatory metabolites are phenylpropanoid biosynthesis-derived compounds, including flavonoids and lignins. Typical groups of flavonoids are shown in the yellow box, several representative groups for specific branches of flavonoid biosynthesis are shown in the deep yellow box, and typical groups for lignins are shown in the light blue box. More information regarding the phenylpropanoid pathway has been reviewed recently [190]. b. R2R3-MYB regulation of glucosinolate biosynthesis in S12 has primarily been studied in Brassicaceae. The synthesis pathway involves three phases, with two types of starting amino acids, that are regulated by different R2R3-MYBs. A fine review with more details has recently been published by Mitreiter and Gigolashvili [191]. c. The best-studied terpenoids regulated by R2R3-MYBs are floral volatiles.

The principal roles of plant *R2R3-MYBs* (~70%) reported to date are in the regulation of plant specialized metabolism, including the benzenoid, phenylpropanoid, terpenoid, and glucosinolate (GSL) pathways (Supplemental Table 2). Nearly half of the functionally characterized *A. thaliana R2R3-MYBs* are related to core and specialized metabolism reactions (Fig. 3 and Supplemental Table 2) [5, 9]. Notably, the majority of these *R2R3-MYBs* are involved as activators or repressors in the transcriptional regulation of the phenylpropanoid biosynthesis pathway, which gives rise to a class of phenylpropanoid-derived compounds such as flavonoids (proanthocyanidins, anthocyanins, flavones, flavonols, isoflavonoids, and phlobaphenes**)**, lignins, and other general phenylpropanoids (Fig. 3a) [43, 77, 189].

Flavonoids are a vast group of plant specialized metabolites with a common diphenylpropane (C6-C3-C6) backbone [45] that have important functions as pigments and/or light protectants. Typical subfamilies in the R2R3-MYB gene family that are involved in the regulation of flavonoid biosynthesis are S6, S5, S4, S7, S44, and S79 (Supplemental Table 2). In addition to their roles in *A. thaliana*, there is substantial emerging evidence for the roles of *R2R3-MYBs* in the transcriptional regulation of flavonoid metabolism in various plants, including important food crops (41), horticultural crops (fruits, 77; ornamentals, 61; vegetables, 37), economically valuable woody crops (27), and edible and medicinal plants (30). With respect to edible crops, including fruits and vegetables, research has mainly focused on substances such as anthocyanins and flavonols that determine their quality and nutritional properties. However, flower colors are key traits for flower crops [42]. Potato *StMYB44* has recently been identified as a repressor of anthocyanin biosynthesis in tuber flesh under high temperatures [192], and whole-genome resequencing-based QTL-seq indicated that *AhTc1* controls the purple testa color in peanut [193]. In addition, the overexpression of *MdMYB24L* resulted in higher anthocyanin contents in transgenic ‘Orin’ apple calli [113]. Similar studies have examined *R2R3-MYBs* in other fruit trees, including *PaMYB10* in apricot [194], *AcMYB123* in kiwifruit [32], *LcMYB5* in litchi [195], and *FcMYB123* in fig [112]. In *M. polymorpha*, *MpMYB14* and *MpMYB02* regulate anthocyanin accumulation [196]. Moreover, different types of flavonoid regulation are broadly observed. For instance, regulatory roles have been reported for *PhMYB15* and *PpMYBF1* in flavonol biosynthesis in peach fruit [197], *FhMYB21L2* in flavonol biosynthesis in *Freesia* [109], *CsPH4* in proanthocyanidin biosynthesis in citrus [198], *SlMYB72* in carotenoid biosynthesis in tomato [199], *CaMYB108* in capsaicinoid biosynthesis in pepper [200], *AgMYB1* in apigenin biosynthesis in celery [137], *HaMYB111* in floral ultraviolet patterning in sunflower [201], and *SmMYB2* in phenolic acid biosynthesis in *Salvia miltiorrhiza* [202]. Apart from model flowers such as *Petunia* and *Antirrhinum*, a number of flowering bulbs have also been studied recently, including *Freesia* [203, 204], *Cattleya* [205], and *Narcissus* [206].

Lignin is another major end-product of the phenylpropanoid pathway and a key component of secondary cell walls (SCWs) in wood; it is polymerized from phenylpropanoid-derived monolignols [207]. The role of *R2R3-MYBs* in the lignin biosynthesis pathway is a major focus of research. As wood is widely used for pulp, papermaking, and biofuels and wood quality is largely determined by lignin synthesis, most studies of the regulatory effects of R2R3-MYBs on lignin synthesis are based on woody plants. For example, the overexpression of *PtoMYB055* upregulates the expression of lignin biosynthetic genes in transgenic poplar, resulting in an increase in the thickness of the SCW [208]. By contrast, *PtMYB189* acts as a repressor to regulate SCW biosynthesis in poplar [209]. Likewise, the positive or negative regulation of the SCW by R2R3-MYBs has also been reported in *Z. mays* [210], *G. hirsutum* [211], *O. sativa* [212], *Pyrus* × *bretschneideri* [213], and *Eriobotrya japonica* [214]; however, additional studies are still needed.

In addition to phenylpropanoid metabolism, which has been investigated extensively in plants, other representative specialized metabolic compounds have been studied, such as those produced by the GSL and terpenoid pathways (Fig. 3b–c). GSLs have well-established anticarcinogenic and antioxidative effects in humans [215]. Interestingly, consistent with the distribution of GSLs, members of S12 are widely distributed in species from the family Brassicaceae and are predominantly related to GSL biosynthesis in this family (Supplemental Table 2). It has been reported that indolic GSL synthesis is regulated mainly by *AtMYB34*, *AtMYB51*, and *AtMYB122*, whereas aliphatic GSL synthesis is regulated by *AtMYB28*, *AtMYB29*, and *AtMYB76* [191]. For example, silencing *BjMYB28* homologs reduces the seed GSL content in *Brassica juncea* [216], *BoMYB29* increases the methylsulphinyl GSL content in *Brassica oleracea* var. *acephala* [217], and *BnMYB28.1* was identified by a QTL analysis using near-isogenic lines [218]. These studies provide good examples of the adaptive evolution of the R2R3-MYB gene family in land plants, especially in angiosperm diversification. Some plants can produce and emit fragrant molecules such as benzenoids and terpenoids, especially in flowers, and these processes are strictly regulated at the transcriptional level by *R2R3-MYBs*. For instance, two *R2R3-MYBs*, *FhMYB21L1* and *FhMYB21L2*, are expressed synchronously with *FhTPS1* and can activate its expression to affect monoterpene synthase synthesis when overexpressed [110]. In addition, *BpMYB21* and *BpMYB61* in *B. platyphylla* are involved in triterpenoid synthesis [219]. *R2R3-MYBs* regulate terpenoid compounds in several medicinal plants, including *S. miltiorrhiza* [220, 221], *Panax ginseng* [222], and *Mentha spicata* [223].

The research summarized above clearly demonstrates that plant *R2R3-MYBs,* which are related to plant specialized metabolism, are mainly involved in the phenylpropanoid biosynthesis pathway, and each subfamily member has key roles in distinct processes, such as proanthocyanidin or lignin biosynthesis. Accordingly, this gene family has probably contributed to functional evolution in land plants.

### The origin and expansion of *R2R3-MYBs* in land plants are associated with functionality

Gene duplication is a prominent event in plant genome evolution and contributes to the establishment of multi-gene families. Genes can be duplicated by various mechanisms, such as whole genome duplication (WGD), chromosomal segmental duplication, tandem duplication, and retrotransposition. Based on systematic analyses of many land plants, we previously found that WGD/polyploidy events and small-scale duplications (e.g. segmental duplications) accounted for the large expansion of the R2R3-MYB gene family [18, 22, 209]. Similar results have been obtained in studies of broad plant lineages [21, 56, 215, 224]. It is well known that WGD events are a driving force in angiosperm diversification. The tremendous expansion of *R2R3-MYBs* in land plants is consistent with WGD in angiosperms, indicating an important role for the expansion of *R2R3-MYBs* in the increased complexity of angiosperms. Small-scale duplications (e.g. tandem/segmental duplications) have also contributed to the rapid expansion and large size of this gene family in land plants, as well as the evolution of novel gene functions [10, 18, 21, 56, 224, 225].

**Figure 4:**
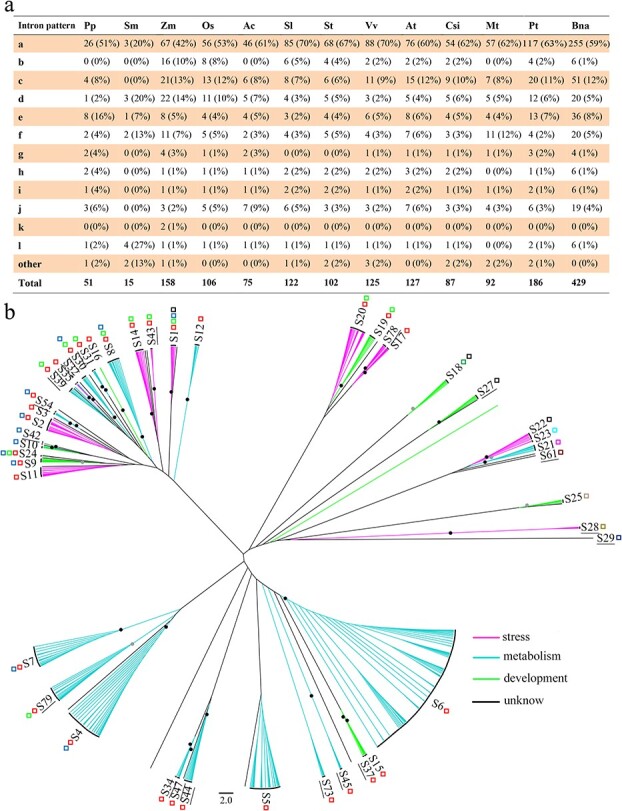
Phylogenetic relationships among functionally characterized plant R2R3-MYBs. a. The number and percentage of *R2R3-MYBs* displaying each intron pattern as shown in Supplemental Fig. 1 in *Physcomitrella patens* (Pp), *Selaginella moellendorfii* (Sm), *Zea mays* (Zm), *Oryza sativa* (Os), *Aquilegia coerulea* (Ac), *Solanum lycopersicum* (Sl), *Solanum tuberosum* (St), *Vitis vinifera* (Vv), *Arabidopsis thaliana* (At), *Citrus sinensis* (Csi), *Medicago truncatula* (Mt), *Populus trichocarpa* (Pt), and *Brassica napus* (Bna). b. The maximum likelihood (ML) tree was constructed with 100 replications using the JJT + G model. It contains 598 nonredundant R2R3-MYBs, including 126 *A. thaliana* R2R3-MYBs [9] and 435 functionally characterized R2R3-MYBs from other plant taxa (Supplemental Table 2), as well as representatives of 73 subfamilies from our previous results [10]. The tree was rooted using S29 (the CDC5-like protein) as the outgroup. The scale bar represents 2 substitutions per site. Detailed information on the ML tree is provided in Supplemental Fig. 2. Detailed information on the sequences used for phylogenetic analyses is provided in Supplemental Table 3. The subfamily classification and nomenclature of the 598 proteins were based on those of *Arabidopsis* [9] and our previous study [10]. The new subfamilies, with the exception of the 25 subfamilies from *Arabidopsis* [9], are underlined, and their detailed functions and classification information are provided in Supplemental Table 2 and Fig. 2. The colored squares indicate the intron pattern to which each subfamily member belongs (Supplemental Fig. 1). Nodes with bootstrap values ≥70% and ≥50% are shown as black and gray dots, respectively, in the phylogenetic tree.

*R2R3-MYBs* underwent a rapid expansion over the course of plant evolution (i.e. from algae to land plants), resulting in a gradual increase in number together with an increase in organismal complexity. Accordingly, the number of genes in this family in land plants is large, showing a huge expansion after the divergence of angiosperms from other vascular plants [10, 60]. For example, only eight *R2R3-MYBs* were detected in the single-celled chlorophyte *Chlamydomonas reinhardtii*, whereas up to 429 *R2R3-MYBs* were observed in the allotetraploid *Brassica napus* [18] (Fig. 1)*.* Based on an analyses of 50 eukaryotes [10], the R2R3-MYB gene family was classified into 73 subfamilies, most of which were newly defined (S26–S73), with the exception of the first 25 subfamilies from *Arabidopsis* [9]. Based on the distribution of subfamily members across the 27 plant genomes investigated in the study [10], it was speculated that a few subfamilies (S18, S21, S22, S26, and S27) were established soon after the origin of land plants and may be the ancestors of land plant *R2R3-MYBs*, whereas the major subfamilies formed a monophyletic clade in land plants [10]. In summary, plant *R2R3-MYBs* experienced three major expansion events: one early in the origin of land plants from Chlorophyta, one after the divergence of spermatophytes from vascular plants, and one in the common ancestor of angiosperms before the divergence of monocots and eudicots, forming the majority of *R2R3-MYB* subfamilies [10, 60]. Notably, members of this gene family exhibit an uneven phylogenetic distribution; the expansion was significantly biased toward subfamilies with three highly homologous intron patterns (a, b, and c) during evolution, resulting in an enormous expansion in the number of *R2R3-MYBs* in the plant lineage [10, 18] (Fig. 4a and Supplemental Fig. 1). For instance, in the 12 land plants investigated in our previous study^10^, *2R-MYBs* with patterns a–c generally accounted for ~66–81% of this gene family in 10 angiosperms, including *Z. mays* (66%), *Arabidopsis* (73%), and *Vitis vinifera* (81%). A similar situation (73%) was found in *B. napus* [18]. By contrast, the corresponding percentages were 59% in *P. patens* and 20% in *S. moellendorffii* [10] (Fig. 4a)*.* Accordingly, up to 43 subfamilies have intron pattern a, and 12 and 19 subfamilies have intron patterns a and b, respectively, and have generally been integrated into the subfamilies with pattern a^10^ (Fig. 4b and Supplemental Fig. 2). Novel subfamilies have commonly been derived from these three categories [10, 18]. By contrast, the numbers of genes with the last nine intron patterns (d–l) are relatively conserved in land plants [10, 18] (Fig. 4a). Relatively few genes have the last nine intron patterns (d–l), which generally account for less than 30% of this gene family in most angiosperms [10]. With the exception of patterns d and j, which are shared by 7 and 2 subfamilies, respectively, each of the last 7 patterns are shared by only one subfamily (e.g. pattern e in S21 and pattern f in S19)^10^ (Fig. 4b and Supplemental Fig. 2). Thus, the number of genes with patterns d–l is relatively conserved in land plants [10, 18].

As discussed above, plant *R2R3-MYBs* are crucial for the regulation of many plant-specific processes related to metabolism (22 subfamilies), development (16 subfamilies), and biotic and abiotic stress processes (14 subfamilies) (Fig. 4b and Supplemental Fig. 2). Notably, the expansion of this gene family is closely accompanied by increases in the functional diversity of *R2R3-MYBs* in the plant kingdom. Interestingly, older subfamilies are generally involved in development and/or stress-related processes, whereas the majority of new/derived subfamilies in angiosperms are related to metabolism (Fig. 4b and Supplemental Fig. 2), suggesting a bias in functional diversification toward specialized metabolism. In particular, the evolution of *R2R3-MYBs* in plants is related to specific expansions giving rise to species- or lineage-specific subfamilies [224]. For example, 5 of 43 *B. napus* subfamilies are Brassicaceae specific [18]. Several of the 43 pineapple subfamilies, such as C2, C8, and C22, are likely to be species or lineage specific [64]. Similarly, three dicot-specific and six grass-specific subfamilies have been observed based on a comparative genomic analysis of *R2R3-MYBs* in *Arabidopsis*, poplar, rice, maize, and switchgrass [226]. Although the functions of most species- or lineage-specific subfamilies are unknown, the distributions suggest that they have lineage-specific functions. For instance, members of the Brassicaceae-specific S12 subfamily (e.g. *AtMYB28/HAG1*, *AtMYB29/HAG3*, *AtMYB34*, *AtMYB51/HIG1*, *AtMYB76/HAG2*, and *AtMYB122* in *A. thaliana*) are regulators of Brassicaceae-specific GSL biosynthesis^5^ (Supplemental Table 2 and Supplemental Fig. 2). Three expanded subfamilies in *Eucalyptus grandis*, including a significantly higher number in woody perennial species (*E. grandis, V. vinifera,* and *P. trichocarpa*), and five subfamilies preferentially found in woody plants are potentially involved in cambium-derived woody growth [224]. Several sugar beet MYBs with an atypical amino acid composition in the R3 domain are species-specific regulators of the betalain red pigment pathway [227, 228]. Together, these results indicate an obvious expansion and functional trend toward specialized metabolism in this gene family during angiosperm evolution.

## Concluding remarks and prospects

*R2R3-MYBs* are ubiquitous in eukaryotes and are not plant specific, but the numbers of this type of MYB gene are generally higher in plants (especially in angiosperms) than in other eukaryotes [10], and they constitute one of the largest TF gene families in plant genomes. As the number of sequenced plant genomes has increased, the plant *R2R3-MYB* gene family has been systematically identified and analyzed in about 74 species at a genome-wide level (Fig. 1). These data have substantially improved our understanding of *R2R3-MYB* distribution, origin, classification, expansion, and evolutionary mechanisms in plants.

As sessile organisms, plants have had to adapt to constantly changing environments during evolution. Through time, plants have differentiated into highly complex organisms and have developed a large number of specialized metabolites with beneficial functions for survival and adaptation. *R2R3-MYBs* have undergone rapid expansion during plant evolution via WGD and small-scale duplications. These drastic expansion/duplication events have played a key role in generating diversity, producing many conserved subfamilies and also lineage-specific subfamilies with specific biological functions (e.g. S12 in GSL biosynthesis), especially those related to specialized metabolism. About 70% of functionally characterized *R2R3-MYBs* and most subfamilies are related to specialized metabolic processes focused on phenylpropanoid-derived secondary metabolites (Fig. 4b and Supplemental Table 2). The rapid expansion and diversification of *R2R3-MYBs* have been ongoing processes throughout the evolution of angiosperms. It is not surprising that this large gene family has contributed to the evolution of physiological or developmental processes that are specific to plants. The expansion of *R2R3-MYBs* in land plants may underpin the emergence of tremendous plant diversity, providing new functional characteristics related to protection against stress and changing environmental conditions. Thus, uncovering the genomic and molecular basis of the origin and evolution of such functional characteristics is a major research goal.

Owing to the availability of resources for gene functional analyses (e.g. genomic data, genetic populations, and experimental tools), a large proportion of the *Arabidopsis R2R3-MYB* gene family has been functionally characterized (about 100 of 126 genes). However, our current knowledge of the functions of plant *R2R3-MYBs* is based mainly on the model plant *Arabidopsis*, whereas studies of non-traditional models (such as crops) are in an early stage. More information about the functions of *R2R3-MYBs* in a broader array of plant taxa will undoubtedly improve our understanding of the mechanisms underlying their evolution and functional diversification. Newly developed genomic technologies (e.g. third-generation long-read sequencing technology) combined with traditional and novel molecular technologies (e.g. genome editing tools) offer excellent opportunities for further improving our understanding of the functions and functional diversification of *R2R3-MYBs*.

## Supplementary Material

Web_Material_uhac058

## References

[1] Klempnauer KH , Gonda TJ, Michael Bishop J. Nucleotide sequence of the retroviral leukemia gene v-MYB and its cellular progenitor c-MYB: the architecture of a transduced oncogene. Cell. 1982;31:453–63. 6297766 10.1016/0092-8674(82)90138-6

[2] Lipsick JS . One billion years of Myb. Oncogene. 1996;13:223–35. 8710361

[3] Rosinski JA , Atchley WR. Molecular evolution of the MYB family of transcription factors: evidence for polyphyletic origin. J Mol Evol. 1998;46:74–83. 9419227 10.1007/pl00006285

[4] Kranz H , Scholz K, Weisshaar B. C-MYB oncogene-like genes encoding three MYB repeats occur in all major plant lineages. Plant J. 2000;21:231–5. 10743663 10.1046/j.1365-313x.2000.00666.x

[5] Dubos C , Stracke R, Grotewold E et al. MYB transcription factors in *Arabidopsis*. Trends Plant Sci. 2010;15:573–81. 20674465 10.1016/j.tplants.2010.06.005

[6] Jiang CK , Rao GY. Insights into the diversification and evolution of R2R3-MYB transcription factors in plants. Plant Physiol. 2020;183:637–55. 32291329 10.1104/pp.19.01082 PMC7271803

[7] Jin J , Zhang H, Kong L et al. PlantTFDB 3.0: a portal for the functional and evolutionary study of plant transcription factors. Nucleic Acids Res. 2014;42:D1182–7. 24174544 10.1093/nar/gkt1016 PMC3965000

[8] Riechmann JL , Heard J, Martin G et al. Arabidopsis transcription factors: genome-wide comparative analysis among eukaryotes. Science. 2000;290:2105–10. 11118137 10.1126/science.290.5499.2105

[9] Stracke R , Werber M, Weisshaar B. The R2R3-MYB gene family in *Arabidopsis thaliana*. Curr Opin Plant Biol. 2001;4:447–56. 11597504 10.1016/s1369-5266(00)00199-0

[10] Du H , Liang Z, Zhao S et al. The evolutionary history of R2R3-MYB proteins across 50 eukaryotes: new insights into subfamily classification and expansion. Sci Rep. 2015;5:11037. 26047035 10.1038/srep11037 PMC4603784

[11] Feller A , Machemer K, Braun EL et al. Evolutionary and comparative analysis of MYB and bHLH plant transcription factors. Plant J. 2011;66:94–116. 21443626 10.1111/j.1365-313X.2010.04459.x

[12] Prouse MB , Campbell MM. The interaction between MYB proteins and their target DNA binding sites. Biochim Biophys Acta. 2012;1819:67–77. 22067744 10.1016/j.bbagrm.2011.10.010

[13] Ogata K , Kanei-Ishii C, Sasaki M et al. The cavity in the hydrophobic core of MYB DNA-binding domain is reserved for DNA recognition and trans-activation. Nat Struct Biol. 1996;3:178–87. 8564545 10.1038/nsb0296-178

[14] Wang H , Wang H, Shao H et al. Recent advances in utilizing transcription factors to improve plant abiotic stress tolerance by transgenic technology. Front Plant Sci. 2016;7:67. 26904044 10.3389/fpls.2016.00067 PMC4746321

[15] Wang B , Luo Q, Li Y et al. Structural insights into target DNA recognition by R2R3-MYB transcription factors. Nucleic Acids Res. 2020;48:460–71. 31733060 10.1093/nar/gkz1081 PMC7145699

[16] Xie Z , Lee E, Lucas JR et al. Regulation of cell proliferation in the stomatal lineage by the *Arabidopsis* MYB FOUR LIPS via direct targeting of core cell cycle genes. Plant Cell. 2010;22:2306–21. 20675570 10.1105/tpc.110.074609 PMC2929110

[17] Jin H , Martin C. Multifunctionality and diversity within the plant MYB-gene family. Plant Mol Biol. 1999;41:577–85. 10645718 10.1023/a:1006319732410

[18] Li P , Wen J, Chen P et al. MYB superfamily in *Brassica napus*: evidence for hormone-mediated expression profiles, large expansion, and functions in root hair development. Biomolecules. 2020;10:875. 10.3390/biom10060875 PMC7356979 32517318

[19] Tombuloglu H . Genome-wide identification and expression analysis of R2R3, 3R-and 4R-MYB transcription factors during lignin biosynthesis in flax (*Linum usitatissimum*). Genomics. 2020;112:782–95. 31128265 10.1016/j.ygeno.2019.05.017

[20] Paz-Ares J , Ghosal D, Wienand U et al. The regulatory c1 locus of *Zea mays* encodes a protein with homology to myb proto-oncogene products and with structural similarities to transcriptional activators. EMBO J. 1987;6:3553–8. 3428265 10.1002/j.1460-2075.1987.tb02684.x PMC553820

[21] Wilkins O , Nahal H, Foong J et al. Expansion and diversification of the Populus R2R3-MYB family of transcription factors. Plant Physiol. 2009;149:981–93. 19091872 10.1104/pp.108.132795 PMC2633813

[22] Du H , Yang SS, Liang Z et al. Genome-wide analysis of the MYB transcription factor superfamily in soybean. BMC Plant Biol. 2012b;12:106. 22776508 10.1186/1471-2229-12-106 PMC3462118

[23] Wang N , Ma Q, Ma J et al. A comparative genome-wide analysis of the R2R3-MYB gene family among four gossypium species and their sequence variation and association with fiber quality traits in an interspecific *G. hirsutum* x *G. barbadense* population. Front Genet. 2019b;10:741. 31475040 10.3389/fgene.2019.00741 PMC6704801

[24] Gocal GF , Poole AT, Gubler F et al. Long-day upregulation of a *GAMYB* gene during *Lolium temulentum* inflorescence formation. Plant Physiol. 1999;119:1271–8. 10198085 10.1104/pp.119.4.1271 PMC32011

[25] Jin H , Cominelli E, Bailey P et al. Transcriptional repression by *AtMYB4* controls production of UV-protecting sunscreens in *Arabidopsis*. EMBO J. 2000;19:6150–61. 11080161 10.1093/emboj/19.22.6150 PMC305818

[26] Jung C , Seo JS, Han SW et al. Overexpression of *AtMYB44* enhances stomatal closure to confer abiotic stress tolerance in transgenic *Arabidopsis*. Plant Physiol. 2008;146:623–35. 18162593 10.1104/pp.107.110981 PMC2245844

[27] Zhang P , Wang R, Ju Q et al. The R2R3-MYB transcription factor MYB49 regulates cadmium accumulation. Plant Physiol. 2019;180:529–42. 30782964 10.1104/pp.18.01380 PMC6501104

[28] Gong Q , Li S, Zheng Y et al. SUMOylation of MYB30 enhances salt tolerance by elevating alternative respiration via transcriptionally upregulating AOX1a in *Arabidopsis*. Plant J. 2020;102:1157–71. 31951058 10.1111/tpj.14689

[29] He J , Liu Y, Yuan D et al. An R2R3 MYB transcription factor confers brown planthopper resistance by regulating the phenylalanine ammonia-lyase pathway in rice. Proc Natl Acad Sci U S A. 2020;117:271–7. 31848246 10.1073/pnas.1902771116 PMC6955232

[30] Stracke R , Ishihara H, Huep G et al. Differential regulation of closely related R2R3-MYB transcription factors controls flavonol accumulation in different parts of the *Arabidopsis thaliana* seedling. Plant J. 2007;50:660–77. 17419845 10.1111/j.1365-313X.2007.03078.x PMC1976380

[31] Tian J , Chen MC, Zhang J et al. Characteristics of dihydroflavonol 4-reductase gene promoters from different leaf colored *malus crabapple* cultivars. Hortic Res. 2017;4:17070. 29263792 10.1038/hortres.2017.70 PMC5727492

[32] Wang L , Tang W, Hu Y et al. A MYB/bHLH complex regulates tissue-specific anthocyanin biosynthesis in the inner pericarp of red-centered kiwifruit *Actinidia chinensis* cv. Plant J. 2019;99:359–78. 30912865 10.1111/tpj.14330

[33] Ding B , Patterson EL, Holalu SV et al. Two MYB proteins in a self-organizing activator-inhibitor system produce spotted pigmentation patterns. Curr Biol. 2020;30:802–814.e8. 32155414 10.1016/j.cub.2019.12.067 PMC7156294

[34] Yan S , Chen N, Huang Z et al. Anthocyanin fruit encodes an R2R3-MYB transcription factor, *SlAN2-like*, activating the transcription of *SlMYBATV* to fine-tune anthocyanin content in tomato fruit. New Phytol. 2020;225:2048–63. 31625612 10.1111/nph.16272

[35] Chen C , Zhang K, Khurshid M et al. MYB transcription repressors regulate plant secondary metabolism. Crit Rev Plant Sci. 2019;38:159–70.

[36] Lee DK , Geisler M, Springer PS. *LATERAL ORGAN FUSION1* and *LATERAL ORGAN FUSION2* function in lateral organ separation and axillary meristem formation in *Arabidopsis*. Development. 2009;136:2423–32. 19542355 10.1242/dev.031971

[37] Mandaokar A , Browse J. MYB108 acts together with MYB24 to regulate jasmonate-mediated stamen maturation in *Arabidopsis*. Plant Physiol. 2009;149:851–62. 19091873 10.1104/pp.108.132597 PMC2633834

[38] Sun W , Gao Z, Wang J et al. Cotton fiber elongation requires the transcription factor *GhMYB212* to regulate sucrose transportation into expanding fibers. New Phytol. 2019;222:864–81. 30506685 10.1111/nph.15620

[39] Baumann K , Perez-Rodriguez M, Bradley D et al. Control of cell and petal morphogenesis by R2R3 MYB transcription factors. Development. 2007;134:1691–701. 17376813 10.1242/dev.02836

[40] Tominaga R , Iwata M, Okada K et al. Functional analysis of the epidermal-specific MYB genes *CAPRICE* and *WEREWOLF* in *Arabidopsis*. Plant Cell. 2007;19:2264–77. 17644729 10.1105/tpc.106.045732 PMC1955706

[41] Yan Y , Li C, Dong X et al. MYB30 is a key negative regulator of *Arabidopsis* photomorphogenic development that promotes PIF4 and PIF5 protein accumulation in the light. Plant Cell. 2020;32:2196–215. 32371543 10.1105/tpc.19.00645 PMC7346557

[42] Naing AH , Kim CK. Roles of R2R3-MYB transcription factors in transcriptional regulation of anthocyanin biosynthesis in horticultural plants. Plant Mol Biol. 2018;98:1–18. 30167900 10.1007/s11103-018-0771-4

[43] Ma D , Constabel CP. MYB repressors as regulators of phenylpropanoid metabolism in plants. Trends Plant Sci. 2019;24:275–89. 30704824 10.1016/j.tplants.2018.12.003

[44] Cao Y , Li K, Li Y et al. MYB transcription factors as regulators of secondary metabolism in plants. Biology (Basel). 2020;9:61. 10.3390/biology9030061 PMC7150910 32213912

[45] Wen W , Alseekh S, Fernie AR. Conservation and diversification of flavonoid metabolism in the plant kingdom. Curr Opin Plant Biol. 2020;55:100–8. 32422532 10.1016/j.pbi.2020.04.004

[46] Yang Y , Liu H. Coordinated shoot and root responses to light signaling in *Arabidopsis*. Plant Commun. 2020;1:100026. 33367230 10.1016/j.xplc.2020.100026 PMC7748005

[47] LaFountain AM , Yuan YW. Repressors of anthocyanin biosynthesis. New Phytol. 2021;231:933–49. 33864686 10.1111/nph.17397 PMC8764531

[48] Grotewold E , Sainz MS, Tagliani L et al. Identification of the residues in the Myb domain of maize C1 that specify the interaction with the bHLH cofactor R. Proc Natl Acad Sci U S A. 2000;97:13579–84. 11095727 10.1073/pnas.250379897 PMC17618

[49] Du H , Feng BR, Yang SS et al. The R2R3-MYB transcription factor gene family in maize. PLoS One. 2012;7:e37463–3. 22719841 10.1371/journal.pone.0037463 PMC3370817

[50] Pu X , Yang L, Liu L et al. Genome-wide analysis of the MYB transcription factor superfamily in *Physcomitrella patens*. Int J Mol Sci. 2020;21:975. 10.3390/ijms21030975 PMC7037163 32024128

[51] Liang Z , Geng Y, Ji C et al. *Mesostigma viride* genome and transcriptome provide insights into the origin and evolution of Streptophyta. Adv Sci. 2020;7:1901850. 10.1002/advs.201901850 PMC6947507 31921561

[52] Li Y , Wang KL, Liu Z et al. Genome-wide analysis and expression profiles of the StR2R3-MYB transcription factor superfamily in potato (*Solanum tuberosum* L.). Int J Biol Macromol. 2020;148:817–32. 31962068 10.1016/j.ijbiomac.2020.01.167

[53] Pucker B . Automatic identification and annotation of MYB gene family members in plants. 2022;23:220. 10.1186/s12864-022-08452-5 PMC8933966 35305581

[54] Millard PS , Kragelund BB, Burow M. R2R3 MYB transcription factors - functions outside the DNA-binding domain. Trends Plant Sci. 2019;24:934–46. 31358471 10.1016/j.tplants.2019.07.003

[55] Sarkar MAR , Watanabe S, Suzuki A et al. Identification of novel MYB transcription factors involved in the isoflavone biosynthetic pathway by using the combination screening system with agroinfiltration and hairy root transformation. Plant Biotechnol. 2019;36:241–51. 10.5511/plantbiotechnology.19.1025a PMC6978502 31983878

[56] Jiang C , Gu X, Peterson T. Identification of conserved gene structures and carboxy-terminal motifs in the MYB gene family of *Arabidopsis* and *Oryza sativa* L. ssp. *indica*. Genome Biol. 2004;5:R46. 15239831 10.1186/gb-2004-5-7-r46 PMC463303

[57] Matus JT , Aquea F, Arce-Johnson P. Analysis of the grape MYB R2R3 subfamily reveals expanded wine quality-related clades and conserved gene structure organization across *Vitis* and *Arabidopsis* genomes. BMC Plant Biol. 2008;8:83–3. 18647406 10.1186/1471-2229-8-83 PMC2507771

[58] Li Q , Zhang C, Li J et al. Genome-wide identification and characterization of R2R3MYB family in *Cucumis sativus*. PLoS One. 2012;7:e47576–6. 23110079 10.1371/journal.pone.0047576 PMC3479133

[59] Bowman JL , Kohchi T, Yamato KT et al. Insights into land plant evolution garnered from the *Marchantia polymorpha* genome. Cell. 2017;171:287–304.e15. 28985561 10.1016/j.cell.2017.09.030

[60] Chang X , Xie S, Wei L et al. Origins and stepwise expansion of R2R3-MYB transcription factors for the terrestrial adaptation of plants. Front Plant Sci. 2020;11:575360. 33424877 10.3389/fpls.2020.575360 PMC7785938

[61] He C , Wang H, Si C et al. Mining MYB transcription factors from the genomes of orchids (*Phalaenopsis* and *dendrobium*) and characterization of an orchid R2R3-MYB gene involved in water-soluble polysaccharide biosynthesis. Sci Rep. 2019;9:13818. 31554868 10.1038/s41598-019-49812-8 PMC6761160

[62] Dias AP , Braun EL, McMullen MD et al. Recently duplicated maize R2R3 MYB genes provide evidence for distinct mechanisms of evolutionary divergence after duplication. Plant Physiol. 2003;131:610–20. 12586885 10.1104/pp.012047 PMC166837

[63] Peng X , Liu H, Wang D et al. Genome-wide identification of the *Jatropha curcas* MYB family and functional analysis of the abiotic stress responsive gene *JcMYB2*. BMC Genomics. 2016;17:251. 27004683 10.1186/s12864-016-2576-7 PMC4804483

[64] Liu C , Xie T, Chen C et al. Genome-wide organization and expression profiling of the R2R3-MYB transcription factor family in pineapple (*Ananas comosus*). BMC Genomics. 2017;18:503. 28668094 10.1186/s12864-017-3896-y PMC5494133

[65] Zhou Q , Jia C, Ma W et al. MYB transcription factors in alfalfa (*Medicago sativa*): genome-wide identification and expression analysis under abiotic stresses. PeerJ. 2019;7:e7714. 31576246 10.7717/peerj.7714 PMC6753925

[66] Zhou W , Zhang Q, Sun Y et al. Genome-wide identification and characterization of R2R3-MYB family in *Hypericum perforatum* under diverse abiotic stresses. Int J Biol Macromol. 2020;145:341–54. 31857171 10.1016/j.ijbiomac.2019.12.100

[67] Li HY , Yue YZ, Ding WJ et al. Genome-wide identification, classification, and expression profiling reveals R2R3-MYB transcription factors related to monoterpenoid biosynthesis in *Osmanthus fragrans*. Genes (Basel). 2020;11:353. 10.3390/genes11040353 PMC7230838 32224874

[68] Carretero-Paulet L , Galstyan A, Roig-Villanova I et al. Genome-wide classification and evolutionary analysis of the bHLH family of transcription factors in Arabidopsis, poplar, Rice, Moss, and algae. Plant Physiol. 2010;153:1398–412. 20472752 10.1104/pp.110.153593 PMC2899937

[69] Vekemans D , Proost S, Vanneste K et al. Gamma paleohexaploidy in the stem lineage of core eudicots: significance for MADS-box gene and species diversification. Mol Biol Evol. 2012;29:3793–806. 22821009 10.1093/molbev/mss183

[70] Cenci A , Rouard M. Evolutionary analyses of GRAS transcription factors in angiosperms. Front Plant Sci. 2017;8:273. 28303145 10.3389/fpls.2017.00273 PMC5332381

[71] Ohta M , Matsui K, Hiratsu K et al. Repression domains of class II ERF transcriptional repressors share an essential motif for active repression. Plant Cell. 2001;13:1959–68. 11487705 10.1105/TPC.010127 PMC139139

[72] Matsui K , Umemura Y, Ohme-Takagi M. AtMYBL2, a protein with a single MYB domain, acts as a negative regulator of anthocyanin biosynthesis in Arabidopsis. Plant J. 2008;55:954–67. 18532977 10.1111/j.1365-313X.2008.03565.x

[73] Rodrigues JA , Espley RV, Allan AC. Genomic analysis uncovers functional variation in the C-terminus of anthocyanin-activating MYB transcription factors. Hortic Res. 2021;8:77. 33790254 10.1038/s41438-021-00514-1 PMC8012628

[74] Chen L , Hu B, Qin Y et al. Advance of the negative regulation of anthocyanin biosynthesis by MYB transcription factors. Plant Physiol Biochem. 2019;136:178–87. 30685697 10.1016/j.plaphy.2019.01.024

[75] Du H , Zhang L, Liu L et al. Biochemical and molecular characterization of plant MYB transcription factor family. Biochemistry (Mosc). 2009;74:1–11. 19232042 10.1134/s0006297909010015

[76] Ambawat S , Sharma P, Yadav NR et al. MYB transcription factor genes as regulators for plant responses: an overview. Physiol Mol Biol Plants. 2013;19:307–21. 24431500 10.1007/s12298-013-0179-1 PMC3715649

[77] Liu J , Osbourn A, Ma P. MYB transcription factors as regulators of phenylpropanoid metabolism in plants. Mol Plant. 2015;8:689–708. 25840349 10.1016/j.molp.2015.03.012

[78] Lee MM , Schiefelbein J. Developmentally distinct MYB genes encode functionally equivalent proteins in *Arabidopsis*. Development. 2001;128:1539–46. 11290293 10.1242/dev.128.9.1539

[79] Higginson T , Li SF, Parish RW. *AtMYB103* regulates tapetum and trichome development in *Arabidopsis thaliana*. Plant J. 2003;35:177–92. 12848824 10.1046/j.1365-313x.2003.01791.x

[80] Oshima Y , Shikata M, Koyoma T et al. MIXTA-like transcription factors and WAX INDUCER1/SHINE1 coordinately regulate cuticle development in *Arabidopsis* and *Torenia fournieri*. Plant Cell. 2013;25:1609–24. 23709630 10.1105/tpc.113.110783 PMC3694695

[81] Shin B , Choi G, Yi H et al. *AtMYB21*, a gene encoding a flower-specific transcription factor, is regulated by COP1. Plant J. 2002;30:23–32. 11967090 10.1046/j.1365-313x.2002.01264.x

[82] Steiner-Lange S , Unte US, Eckstein L et al. Disruption of *Arabidopsis thaliana* MYB26 results in male sterility due to non-dehiscent anthers. Plant J. 2003;34:519–28. 12753590 10.1046/j.1365-313x.2003.01745.x

[83] Li J , Chen T, Huang F et al. Ectopic expression of a R2R3 MYB transcription factor of dove tree (*Davidia involucrata*) aggravates seed abortion in *Arabidopsis thaliana*. Funct Plant Biol. 2020;47:454–63. 32213273 10.1071/FP19317

[84] Li SF , Allen PJ, Napoli RS et al. MYB-bHLH-TTG1 regulates *Arabidopsis* seed coat biosynthesis pathways directly and indirectly via multiple tiers of transcription factors. Plant Cell Physiol. 2020;61:1005–18. 32154880 10.1093/pcp/pcaa027

[85] Tan H , Man C, Xie Y et al. A crucial role of GA-regulated flavonol biosynthesis in root growth of *Arabidopsis*. Mol Plant. 2019;12:521–37. 30630075 10.1016/j.molp.2018.12.021

[86] Wang W , Ryu KH, Barron C et al. Root epidermal cell patterning is modulated by a critical residue in the WEREWOLF transcription factor. Plant Physiol. 2019;181:1239–56. 31492737 10.1104/pp.19.00458 PMC6836813

[87] Xu B , Taylor L, Pucker B et al. The land plant-specific MIXTA-MYB lineage is implicated in the early evolution of the plant cuticle and the colonization of land. New Phytol. 2021;229:2324–38. 33051877 10.1111/nph.16997

[88] Perez-Rodriguez M , Jaffe FW, Butelli E et al. 2005 development of three different cell types is associated with the activity of a specific MYB transcription factor in the ventral petal of *Antirrhinum majus* flowers. Development. 2005;132:359–70. 15604096 10.1242/dev.01584

[89] Zhou C , Li C. A novel R2R3-MYB transcription factor *BpMYB106* of birch (*Betula platyphylla*) confers increased photosynthesis and growth rate through upregulating photosynthetic gene expression. Front Plant Sci. 2016;7:315. 27047502 10.3389/fpls.2016.00315 PMC4801893

[90] Wu H , Tian Y, Wan Q et al. Genetics and evolution of MIXTA genes regulating cotton lint fiber development. New Phytol. 2018;217:883–95. 29034968 10.1111/nph.14844

[91] Millar AA , Gubler F. The *Arabidopsis GAMYB-like* genes, *MYB33* and *MYB65*, are microRNA-regulated genes that redundantly facilitate anther development. Plant Cell. 2005;17:705–21. 15722475 10.1105/tpc.104.027920 PMC1069693

[92] Shen X , Hu Z, Xiang X et al. Overexpression of a stamen-specific R2R3-MYB gene *BcMF28* causes aberrant stamen development in transgenic *Arabidopsis*. Biochem Biophys Res Commun. 2019;518:726–31. 31472956 10.1016/j.bbrc.2019.08.119

[93] Aya K , Ueguchi-Tanaka M, Kondo M et al. Gibberellin modulates anther development in rice via the transcriptional regulation of GAMYB. Plant Cell. 2009;21:1453–72. 19454733 10.1105/tpc.108.062935 PMC2700530

[94] Liang Y , Tan ZM, Zhu L et al. *MYB97*, *MYB101* and *MYB120* function as male factors that control pollen tube-synergid interaction in *Arabidopsis thaliana* fertilization. PLoS Genet. 2013;9:e1003933. 24278028 10.1371/journal.pgen.1003933 PMC3836714

[95] Rabiger DS , Drews GN. MYB64 and MYB119 are required for cellularization and differentiation during female gametogenesis in *Arabidopsis thaliana*. PLoS Genet. 2013;9:e1003783. 24068955 10.1371/journal.pgen.1003783 PMC3778002

[96] Hisanaga T , Okahashi K, Yamaoka S et al. A cis-acting bidirectional transcription switch controls sexual dimorphism in the liverwort. EMBO J. 2019;38:e100240. 30609993 10.15252/embj.2018100240 PMC6418429

[97] Aya K , Hiwatashi Y, Kojima M et al. The gibberellin perception system evolved to regulate a pre-existing GAMYB-mediated system during land plant evolution. Nat Commun. 2011;2:544. 22109518 10.1038/ncomms1552

[98] Huang C , Hu G, Li F et al. *NbPHAN*, a MYB transcriptional factor, regulates leaf development and affects drought tolerance in *Nicotiana benthamiana*. Physiol Plant. 2013;149:297–309. 23387304 10.1111/ppl.12031

[99] Ge L , Chen R. PHANTASTICA regulates leaf polarity and petiole identity in *Medicago truncatula*. Plant Signal Behav. 2014;9:e28121–1. 24603499 10.4161/psb.28121 PMC4091575

[100] Tsiantis M , Schneeberger R, Golz JF et al. The maize rough sheath2 gene and leaf development programs in monocot and dicot plants. Science. 1999;284:154–6. 10102817 10.1126/science.284.5411.154

[101] Morimoto R , Nishioka E, Murai K et al. Functional conservation of wheat orthologs of maize *rough sheath1* and *rough sheath2* genes. Plant Mol Biol. 2009;69:273–85. 18974935 10.1007/s11103-008-9422-5

[102] Zoulias N , Koenig D, Hamidi A et al. A role for PHANTASTICA in medio-lateral regulation of adaxial domain development in tomato and tobacco leaves. Ann Bot. 2012;109:407–18. 22184618 10.1093/aob/mcr295 PMC3268540

[103] Hong SH , Kim HJ, Ryu JS et al. CRY1 inhibits COP1-mediated degradation of BIT1, a MYB transcription factor, to activate blue light-dependent gene expression in *Arabidopsis*. Plant J. 2008;55:361–71. 18397371 10.1111/j.1365-313X.2008.03508.x

[104] Fernández-Marcos M , Desvoyes B, Manzano C et al. Control of *Arabidopsis* lateral root primordium boundaries by MYB36. New Phytol. 2017;213:105–12. 27891649 10.1111/nph.14304 PMC5126979

[105] Yasui Y , Tsukamoto S, Sugaya T et al. GEMMA CUP-ASSOCIATED MYB1, an ortholog of axillary meristem regulators, is essential in vegetative reproduction in *Marchantia polymorpha*. Curr Biol. 2019;29:3987–3995.e5. 31708390 10.1016/j.cub.2019.10.004

[106] Leech MJ , Kammerer W, Cove DJ et al. Expression of myb-related genes in the moss. Plant J. 1993;3:51–61. 8401607 10.1046/j.1365-313x.1993.t01-3-00999.x

[107] Mandaokar A , Thines B, Shin B et al. Transcriptional regulators of stamen development in Arabidopsis identified by transcriptional profiling. Plant J. 2006;46:984–1008. 16805732 10.1111/j.1365-313X.2006.02756.x

[108] Cheng H , Song S, Xiao L et al. Gibberellin acts through jasmonate to control the expression of MYB21, MYB24, and MYB57 to promote stamen filament growth in *Arabidopsis*. PLoS Genet. 2009;5:e1000440. 19325888 10.1371/journal.pgen.1000440 PMC2654962

[109] Shan X , Li Y, Yang S et al. The spatio-temporal biosynthesis of floral flavonols is controlled by differential phylogenetic MYB regulators in *Freesia hybrida*. New Phytol. 2020;228:1864–79. 32696979 10.1111/nph.16818

[110] Yang Z , Li Y, Gao F et al. MYB21 interacts with MYC2 to control the expression of terpene synthase genes in flowers of *Freesia hybrida* and *Arabidopsis thaliana*. J Exp Bot. 2020;71:4140–58. 32275056 10.1093/jxb/eraa184

[111] Nakatsuka T , Haruta KS, Abe Y et al. Identification and characterization of R2R3-MYB and bHLH transcription factors regulating anthocyanin biosynthesis in gentian flowers. Plant Cell Physiol. 2008;49:1818–29. 18974195 10.1093/pcp/pcn163

[112] Li J , An Y, Wang L. Transcriptomic analysis of *Ficus carica* peels with a focus on the key genes for anthocyanin biosynthesis. Int J Mol Sci. 2020;21:1245. 10.3390/ijms21041245 PMC7072940 32069906

[113] Wang Y , Liu W, Jiang H et al. The R2R3-MYB transcription factor *MdMYB24-like* is involved in methyl jasmonate-induced anthocyanin biosynthesis in apple. Plant Physiol Biochem. 2019;139:273–82. 30925437 10.1016/j.plaphy.2019.03.031

[114] Spitzer-Rimon B , Marhevka E, Barkai O et al. *EOBII*, a gene encoding a flower-specific regulator of phenylpropanoid volatiles' biosynthesis in petunia. Plant Cell. 2010;22:1961–76. 20543029 10.1105/tpc.109.067280 PMC2910970

[115] Yang SW , Jang IC, Henriques R et al. FAR-RED ELONGATED HYPOCOTYL1 and FHY1-LIKE associate with the *Arabidopsis* transcription factors LAF1 and HFR1 to transmit phytochrome a signals for inhibition of HYPOCOTYL elongation. Plant Cell. 2009;21:1341–59. 19482971 10.1105/tpc.109.067215 PMC2700525

[116] Gibbs DJ , Vob U, Harding SA et al. *AtMYB93* is a novel negative regulator of lateral root development in *Arabidopsis*. New Phytol. 2014;203:1194–207. 24902892 10.1111/nph.12879 PMC4286813

[117] Wang Z , Li J, Mao Y et al. Transcriptional regulation of MdPIN3 and MdPIN10 by MdFLP during apple self-rooted stock adventitious root gravitropism. BMC Plant Biol. 2019;19. 10.1186/s12870-019-1847-2 PMC6543673 31146692

[118] Yuan Y , Xu X, Luo Y et al. R2R3 MYB-dependent auxin signalling regulates trichome formation, and increased trichome density confers spider mite tolerance on tomato. Plant Biotechnol J. 2021;19:138–52. 32654333 10.1111/pbi.13448 PMC7769234

[119] Zhu J , Chen H, Li H et al. Defective in tapetal development and function 1 is essential for anther development and tapetal function for microspore maturation in *Arabidopsis*. Plant J. 2008;55:266–77. 18397379 10.1111/j.1365-313X.2008.03500.x

[120] Zhang Y , Cao G, Qu LJ et al. Characterization of *Arabidopsis* MYB transcription factor gene *AtMYB17* and its possible regulation by LEAFY and AGL15. J Genet Genomics. 2009;36:99–107. 19232308 10.1016/S1673-8527(08)60096-X

[121] Liang G , He H, Li Y et al. MYB82 functions in regulation of trichome development in Arabidopsis. J Exp Bot. 2014;65:3215–23. 24803498 10.1093/jxb/eru179 PMC4071844

[122] Zhang Y , Zhu H, Shao C et al. *PaMYB82* from *Platanus acerifolia* regulates trichome development in transgenic *Arabidopsis*. Plant Sci. 2019;287:110177. 31481209 10.1016/j.plantsci.2019.110177

[123] Yang A , Dai X, Zhang WH. A R2R3-type MYB gene, *OsMYB2*, is involved in salt, cold, and dehydration tolerance in rice. J Exp Bot. 2012;63:2541–56. 22301384 10.1093/jxb/err431 PMC3346221

[124] Zhao Y , Cheng X, Liu X et al. The wheat MYB transcription factor *TaMYB(31)* is involved in drought stress responses in *Arabidopsis*. Front Plant Sci. 2018;9:1426–6. 30323824 10.3389/fpls.2018.01426 PMC6172359

[125] Agarwal P , Mitra M, Banerjee S et al. MYB4 transcription factor, a member of R2R3-subfamily of MYB domain protein, regulates cadmium tolerance via enhanced protection against oxidative damage and increases expression of *PCS1* and *MT1C* in *Arabidopsis*. Plant Sci. 2020;297:110501. 32563471 10.1016/j.plantsci.2020.110501

[126] Fichman Y , Zandalinas SI, Sengupta S et al. MYB30 orchestrates systemic reactive oxygen signaling and plant acclimation. Plant Physiol. 2020;184:666. 32699028 10.1104/pp.20.00859 PMC7536697

[127] Yao L , Yang B, Xian B et al. The R2R3-MYB transcription factor *BnaMYB111L* from rapeseed modulates reactive oxygen species accumulation and hypersensitive-like cell death. Physiologie Végétale. 2020;147:280–8. 10.1016/j.plaphy.2019.12.027 31891862

[128] Li C , Ng CKY, Fan LM. MYB transcription factors, active players in abiotic stress signaling. Environ Exp Bot. 2015;114:80–91.

[129] Ng DWK , Abeysinghe JK, Kamali M. Regulating the regulators: the control of transcription factors in plant defense signaling. Int J Mol Sci. 2018;19:3737. 10.3390/ijms19123737 PMC6321093 30477211

[130] Cominelli E , Galbiati M, Vavassuer A et al. A guard-cell-specific MYB transcription factor regulates stomatal movements and plant drought tolerance. Curr Biol. 2005;15:1196–200. 16005291 10.1016/j.cub.2005.05.048

[131] Seo PJ , Xiang F, Qiao M et al. The MYB96 transcription factor mediates abscisic acid signaling during drought stress response in *Arabidopsis*. Plant Physiol. 2009;151:275–89. 19625633 10.1104/pp.109.144220 PMC2735973

[132] Seo PJ , Lee SB, Chung M et al. The MYB96 transcription factor regulates cuticular wax biosynthesis under drought conditions in *Arabidopsis*. Plant Cell. 2011;23:1138–52. 21398568 10.1105/tpc.111.083485 PMC3082259

[133] Marino D , Froidure S, Canonne J et al. *Arabidopsis* ubiquitin ligase MIEL1 mediates degradation of the transcription factor MYB30 weakening plant defence. Nat Commun. 2013;4:1476. 23403577 10.1038/ncomms2479

[134] Rusconi F , Simeoni F, Francia P et al. The *Arabidopsis thaliana* MYB60 promoter provides a tool for the spatio-temporal control of gene expression in stomatal guard cells. J Exp Bot. 2013;64:3361–71. 23828545 10.1093/jxb/ert180 PMC3733157

[135] Lee HG , Seo PJ. The MYB96–HHP module integrates cold and abscisic acid signaling to activate the CBF–COR pathway in *Arabidopsis*. Plant J. 2015;82:962–77. 25912720 10.1111/tpj.12866

[136] Liao C , Zheng Y, Guo Y. MYB30 transcription factor regulates oxidative and heat stress responses through ANNEXIN-mediated cytosolic calcium signaling in *Arabidopsis*. New Phytol. 2017;216:163–77. 28726305 10.1111/nph.14679

[137] Yan J , Yu L, He L et al. Comparative transcriptome analysis of celery leaf blades identified an R2R3-MYB transcription factor that regulates apigenin metabolism. J Agric Food Chem. 2019;67:5265–77. 30969771 10.1021/acs.jafc.9b01052

[138] Kirik V , Kölle K, Miséra S et al. Two novel MYB homologues with changed expression in late embryogenesis-defective *Arabidopsis* mutants. Plant Mol Biol. 1998;37:819–27. 9678577 10.1023/a:1006011002499

[139] Ding Z , Li S, An X et al. Transgenic expression of *MYB15* confers enhanced sensitivity to abscisic acid and improved drought tolerance in *Arabidopsis thaliana*. J Genet Genomics. 2009;36:17–29. 19161942 10.1016/S1673-8527(09)60003-5

[140] Chen Y , Chen Z, Kang J et al. *AtMYB14* regulates cold tolerance in *Arabidopsis*. *Plant Mol. Biol*. Report. 2013;31:87–97. 10.1007/s11105-012-0481-z PMC3881570 24415840

[141] Chezem WR , Memon A, Li FS et al. SG2-type R2R3-MYB transcription factor MYB15 controls defense-induced lignification and basal immunity in *Arabidopsis*. Plant Cell. 2017;29:1907–26. 28733420 10.1105/tpc.16.00954 PMC5590497

[142] Hoang MHT , Nguyen XC, Lee K et al. Phosphorylation by *AtMPK6* is required for the biological function of *AtMYB41* in Arabidopsis. Biochem Biophys Res Commun. 2012;422:181–6. 22575450 10.1016/j.bbrc.2012.04.137

[143] Xu R , Wang Y, Zheng H et al. Salt-induced transcription factor MYB74 is regulated by the RNA-directed DNA methylation pathway in *Arabidopsis*. J Exp Bot. 2015;66:5997–6008. 26139822 10.1093/jxb/erv312 PMC4566987

[144] Devaiah BN , Madhuvanthi R, Karthikeyan AS et al. Phosphate starvation responses and gibberellic acid biosynthesis are regulated by the MYB62 transcription factor in *Arabidopsis*. Mol Plant. 2009;2:43–58. 19529828 10.1093/mp/ssn081 PMC2639739

[145] Baek D , Kim MC, Chun HJ et al. Regulation of miR399f transcription by *AtMYB2* affects phosphate starvation responses in *Arabidopsis*. Plant Physiol. 2013;161:362–73. 23154535 10.1104/pp.112.205922 PMC3532267

[146] Chou ML , Liao WY, Wei WC et al. The direct involvement of dark-induced Tic55 protein in chlorophyll catabolism and its indirect role in the MYB108-NAC signaling pathway during leaf senescence in *Arabidopsis thaliana*. Int J Mol Sci. 2018;19:1854. 10.3390/ijms19071854 PMC6073118 29937503

[147] Zhao Y , Xing L, Wang X et al. The ABA receptor PYL8 promotes lateral root growth by enhancing MYB77-dependent transcription of auxin-responsive genes. Sci Signal. 2014;7:ra53. 24894996 10.1126/scisignal.2005051 PMC4298826

[148] Nguyen NH , Cheong JJ. The *AtMYB44* promoter is accessible to signals that induce different chromatin modifications for gene transcription. Plant Physiol Biochem. 2018;130:14–9. 29957571 10.1016/j.plaphy.2018.06.030

[149] Nishida S , Kakei Y, Shimada Y et al. Genome-wide analysis of specific alterations in transcript structure and accumulation caused by nutrient deficiencies in *Arabidopsis thaliana*. Plant J. 2017;91:741–53. 28586097 10.1111/tpj.13606

[150] Palmer CM , Hindt MN, Schmidt H et al. MYB10 and MYB72 are required for growth under iron-limiting conditions. PLoS Genet. 2013;9:e1003953. 24278034 10.1371/journal.pgen.1003953 PMC3836873

[151] Cui MH , Shin-Yoo K, Hyoung S et al. An *Arabidopsis* R2R3-MYB transcription factor, *AtMYB20*, negatively regulates type 2C serine/threonine protein phosphatases to enhance salt tolerance. FEBS Lett. 2013;587:1773–8. 23660402 10.1016/j.febslet.2013.04.028

[152] Wang T , Tohge T, Ivakov A et al. Salt-related MYB1 coordinates abscisic acid biosynthesis and signaling during salt stress in *Arabidopsis*. Plant Physiol. 2015;169:1027–41. 26243618 10.1104/pp.15.00962 PMC4587467

[153] Agarwal M , Hao Y, Kapoor A et al. A R2R3 type MYB transcription factor is involved in the cold regulation of CBF genes and in acquired freezing tolerance. J Biol Chem. 2006;281:37636–45. 17015446 10.1074/jbc.M605895200

[154] He Y , Li W, Lv J et al. Ectopic expression of a wheat MYB transcription factor gene, *TaMYB73*, improves salinity stress tolerance in *Arabidopsis thaliana*. J Exp Bot. 2012;63:1511–22. 22140235 10.1093/jxb/err389

[155] Chen S , Wu F, Li Y et al. *NtMYB4* and *NtCHS1* are critical factors in the regulation of flavonoid biosynthesis and are involved in salinity responsiveness. Front Plant Sci. 2019;10:178. 30846995 10.3389/fpls.2019.00178 PMC6393349

[156] Liao W , Yang Y, Li Y et al. Genome-wide identification of cassava R2R3 MYB family genes related to abscission zone separation after environmental-stress-induced abscission. Sci Rep. 2016;6:32006–6. 27573926 10.1038/srep32006 PMC5004182

[157] Fang Q , Jiang T, Xu L et al. A salt-stress-regulator from the poplar R2R3 MYB family integrates the regulation of lateral root emergence and ABA signaling to mediate salt stress tolerance in *Arabidopsis*. Plant Physiol Biochem. 2017;114:100–10. 28285084 10.1016/j.plaphy.2017.02.018

[158] Zhang X , Chen L, Shi Q et al. *SIMYB102*, an R2R3-type MYB gene, confers salt tolerance in transgenic tomato. Plant Sci. 2020;291:110356. 31928668 10.1016/j.plantsci.2019.110356

[159] Zhang Z , Liu X, Wang X et al. An R2R3 MYB transcription factor in wheat, *TaPIMP1*, mediates host resistance to *Bipolaris sorokiniana* and drought stresses through regulation of defense- and stress-related genes. New Phytol. 2012;196:1155–70. 23046089 10.1111/j.1469-8137.2012.04353.x

[160] Zhang Z , Hu X, Zhang Y et al. Opposing control by transcription factors MYB61 and MYB3 increases freezing tolerance by relieving C-repeat binding factor suppression. Plant Physiol. 2016;172:1306–23. 27578551 10.1104/pp.16.00051 PMC5047070

[161] Zhang S , Zhao Q, Zeng D et al. *RhMYB108*, an R2R3-MYB transcription factor, is involved in ethylene- and JA-induced petal senescence in rose plants. Hortic Res. 2019;6:131. 31814984 10.1038/s41438-019-0221-8 PMC6885062

[162] Shukla PS , Agarwal P, Gupta K et al. Molecular characterization of an MYB transcription factor from a succulent halophyte involved in stress tolerance. Aob Plants. 2015;7:plv054. 25986050 10.1093/aobpla/plv054 PMC4497479

[163] Gao F , Zhao HX, Yao HP et al. Identification, isolation and expression analysis of eight stress-related R2R3-MYB genes in tartary buckwheat (*Fagopyrum tataricum*). Plant Cell Rep. 2016;35:1385–96. 27021383 10.1007/s00299-016-1971-5

[164] Hu DG , Li YY, Zhang QY et al. The R2R3-MYB transcription factor *MdMYB73* is involved in malate accumulation and vacuolar acidification in apple. Plant J. 2017;91:443–54. 28423209 10.1111/tpj.13579

[165] Zhao Y , Yang Z, Ding Y et al. Over-expression of an R2R3 MYB gene, *GhMYB73*, increases tolerance to salt stress in transgenic *Arabidopsis*. Plant Sci. 2019;286:28–36. 31300139 10.1016/j.plantsci.2019.05.021

[166] Lee TG , Jang CS, Kim JY et al. A MYB transcription factor (*TaMyb1*) from wheat roots is expressed during hypoxia: roles in response to the oxygen concentration in root environment and abiotic stresses. Physiol Plant. 2007;129:375–85.

[167] Garg B , Lata C, Prasad M. A study of the role of gene TaMYB2 and an associated SNP in dehydration tolerance in common wheat. Mol Biol Rep. 2012;39:10865–71. 23065204 10.1007/s11033-012-1983-3

[168] Cao ZH , Zhang SZ, Wang RK et al. Genome wide analysis of the apple myb transcription factor family allows the identification of *MdoMYB121* gene confering abiotic stress tolerance in plants. PLoS One. 2013;8:e69955. 23950843 10.1371/journal.pone.0069955 PMC3735319

[169] Wang RK , Cao ZH, Hao YJ. Overexpression of a R2R3 MYB gene *MdSIMYB1* increases tolerance to multiple stresses in transgenic tobacco and apples. Physiol Plant. 2014;150:76–87. 23647377 10.1111/ppl.12069

[170] Zhang L , Liu G, Zhao G et al. Characterization of a wheat R2R3-MYB transcription factor gene, *TaMYB19*, involved in enhanced abiotic stresses in *Arabidopsis*. Plant Cell Physiol. 2014;55:1802–12. 25146486 10.1093/pcp/pcu109

[171] Dong W , Song Y, Zhao Z et al. The *Medicago truncatula* R2R3-MYB transcription factor gene *MtMYBS1* enhances salinity tolerance when constitutively expressed in *Arabidopsis thaliana*. Biochem Biophys Res Commun. 2017;490:225–30. 28602696 10.1016/j.bbrc.2017.06.025

[172] Wei H , Zhao H, Su T et al. Chicory R2R3-MYB transcription factors *CiMYB5* and *CiMYB3* regulate fructan 1-exohydrolase expression in response to abiotic stress and hormonal cues. J Exp Bot. 2017;68:4323–38. 28922763 10.1093/jxb/erx210 PMC5853547

[173] Geng D , Chen P, Shen X et al. *MdMYB88* and *MdMYB124* enhance drought tolerance by modulating root vessels and cell walls in apple. Plant Physiol. 2018;178:1296–309. 30190418 10.1104/pp.18.00502 PMC6236628

[174] He Y , Yang X, Xu C et al. Overexpression of a novel transcriptional repressor *GmMYB3a* negatively regulates salt-alkali tolerance and stress-related genes in soybean. Biochem Biophys Res Commun. 2018;498:586–91. 29524418 10.1016/j.bbrc.2018.03.026

[175] Xie Y , Chen P, Yan Y et al. An atypical R2R3 MYB transcription factor increases cold hardiness by CBF-dependent and CBF-independent pathways in apple. New Phytol. 2018;218:201–18. 29266327 10.1111/nph.14952

[176] Meng C , Sui N. Overexpression of maize MYB-IF35 increases chilling tolerance in Arabidopsis. Physiologie Végétale. 2019;135:167–73. 10.1016/j.plaphy.2018.11.038 30553138

[177] Xing C , Liu Y, Zhao L et al. A novel MYB transcription factor regulates ascorbic acid synthesis and affects cold tolerance. Plant Cell Environ. 2019;42:832–45. 29929211 10.1111/pce.13387

[178] He Y , Dong Y, Yang X et al. Functional activation of a novel R2R3-MYB protein gene, *GmMYB68*, confers salt-alkali resistance in soybean (*Glycine max* L.). Genome. 2020b;63:13–26. 31550433 10.1139/gen-2018-0132

[179] An C , Sheng L, Du X et al. Overexpression of *CmMYB15* provides chrysanthemum resistance to aphids by regulating the biosynthesis of lignin. Hortic Res. 2019;6:84. 31645945 10.1038/s41438-019-0166-y PMC6804602

[180] Jahan MA , Harris B, Lowery M et al. Glyceollin transcription factor *GmMYB29A2* regulates soybean resistance to *Phytophthora sojae*. Plant Physiol. 2020;183:530–46. 32209590 10.1104/pp.19.01293 PMC7271783

[181] Yu Y , Guo D, Li G et al. The grapevine R2R3-type MYB transcription factor *VdMYB1* positively regulates defense responses by activating the stilbene synthase gene 2 (VdSTS2). BMC Plant Biol. 2019;19:478. 31699028 10.1186/s12870-019-1993-6 PMC6836392

[182] Shan T , Rong W, Xu H et al. The wheat R2R3-MYB transcription factor *TaRIM1* participates in resistance response against the pathogen *Rhizoctonia cerealis* infection through regulating defense genes. Sci Rep. 2016;6:28777. 27364458 10.1038/srep28777 PMC4929490

[183] Raffaele S , Vailleau F, Leger A et al. A MYB transcription factor regulates very-long-chain fatty acid biosynthesis for activation of the hypersensitive cell death response in *Arabidopsis*. Plant Cell. 2008;20:752–67. 18326828 10.1105/tpc.107.054858 PMC2329921

[184] Seo PJ , Park CM. MYB96-mediated abscisic acid signals induce pathogen resistance response by promoting salicylic acid biosynthesis in *Arabidopsis*. New Phytol. 2010;186:471–83. 20149112 10.1111/j.1469-8137.2010.03183.x

[185] Zhu L , Guo J, Ma Z et al. *Arabidopsis* transcription factor MYB102 increases plant susceptibility to aphids by substantial activation of ethylene biosynthesis. Biomol Ther. 2018;8:39. 10.3390/biom8020039 PMC6023100 29880735

[186] Segarra G , Van der Ent S, Trillas I et al. MYB72, a node of convergence in induced systemic resistance triggered by a fungal and a bacterial beneficial microbe. Plant Biol. 2009;11:90–6. 19121118 10.1111/j.1438-8677.2008.00162.x

[187] Stringlis IA , Yu K, Fuessner K et al. MYB72-dependent coumarin exudation shapes root microbiome assembly to promote plant health. Proc Natl Acad Sci U S A. 2018;115:E5213–22. 29686086 10.1073/pnas.1722335115 PMC5984513

[188] Lee MW , Qi M, Yang Y. A novel jasmonic acid-inducible rice MYB gene associates with fungal infection and host cell death. Mol Plant-Microbe Interact. 2001;14:527–35. 11310740 10.1094/MPMI.2001.14.4.527

[189] Deng Y , Lu S. Biosynthesis and regulation of phenylpropanoids in plants. Front Plant Sci. 2017;36:257–90.

[190] Dong NQ , Lin HX. Contribution of phenylpropanoid metabolism to plant development and plant-environment interactions. J Integr Plant Biol. 2021;63:180–209. 33325112 10.1111/jipb.13054

[191] Mitreiter S , Gigolashvili T. Regulation of glucosinolate biosynthesis. J Exp Bot. 2021;72:70–91. 33313802 10.1093/jxb/eraa479

[192] Liu Y , Wang KL, Espley RV et al. *StMYB44* negatively regulates anthocyanin biosynthesis at high temperatures in tuber flesh of potato. J Exp Bot. 2019;70:3809–24. 31020330 10.1093/jxb/erz194 PMC6685667

[193] Zhao Y , Ma J, Li M et al. Whole-genome resequencing-based QTL-seq identified *AhTc1* gene encoding a R2R3-MYB transcription factor controlling peanut purple testa colour. Plant Biotechnol J. 2020;18:96–105. 31131506 10.1111/pbi.13175 PMC6920131

[194] Xi W , Feng J, Liu Y et al. The R2R3-MYB transcription factor PaMYB10 is involved in anthocyanin biosynthesis in apricots and determines red blushed skin. BMC Plant Biol. 2019;19. 10.1186/s12870-019-1898-4 PMC6604168 31262258

[195] Lai B , Du LN, Hu B et al. Characterization of a novel litchi R2R3-MYB transcription factor that involves in anthocyanin biosynthesis and tissue acidification. BMC Plant Biol. 2019;19:62. 30732564 10.1186/s12870-019-1658-5 PMC6367832

[196] Kubo H , Nozawa S, Hiwatashi T et al. Biosynthesis of riccionidins and marchantins is regulated by R2R3-MYB transcription factors in *Marchantia polymorpha*. J Plant Res. 2018;131:849–64. 29845372 10.1007/s10265-018-1044-7

[197] Cao Y , Xie L, Ma Y et al. *PpMYB15* and *PpMYBF1* transcription factors are involved in regulating flavonol biosynthesis in peach fruit. J Agric Food Chem. 2019;67:644–52. 30525549 10.1021/acs.jafc.8b04810

[198] Zhang Y , Ye J, Liu C et al. Citrus PH4-Noemi regulatory complex is involved in proanthocyanidin biosynthesis via a positive feedback loop. J Exp Bot. 2020;71:1306–21. 31728522 10.1093/jxb/erz506 PMC7031078

[199] Wu M , Xu X, Hu X et al. *SlMYB72* regulates the metabolism of chlorophylls, carotenoids, and flavonoids in tomato fruit. Plant Physiol. 2020;183:854–68. 32414899 10.1104/pp.20.00156 PMC7333684

[200] Sun B , Zhu Z, Chen C et al. Jasmonate-inducible R2R3-MYB transcription factor regulates capsaicinoid biosynthesis and stamen development in *capsicum*. J Agric Food Chem. 2019;67:10891–903. 31505929 10.1021/acs.jafc.9b04978

[201] Todesco M , Bercovich N, Kim A et al. Genetic basis and dual adaptive role of floral pigmentation in sunflowers. Elife. 2022;11:e72072. 35040432 10.7554/eLife.72072 PMC8765750

[202] Deng C , Wang Y, Huang F et al. *SmMYB2* promotes salvianolic acid biosynthesis in the medicinal herb *Salvia miltiorrhiza*. J Integr Plant Biol. 2020;62:1688–702. 32343491 10.1111/jipb.12943

[203] Li Y , Shan X, Zhou L et al. The R2R3-MYB factor *FhMYB5* from *Freesia hybrida* contributes to the regulation of anthocyanin and proanthocyanidin in biosynthesis. Front Plant Sci. 2019;9:1935. 30666265 10.3389/fpls.2018.01935 PMC6330306

[204] Li Y , Shan X, Tong L et al. The conserved and particular roles of R2R3-MYB regulator *FhPAP1* from *Freesia hybrida* in flower anthocyanin biosynthesis. Plant Cell Physiol. 2020;61:1365–80. 32392327 10.1093/pcp/pcaa065

[205] Li BJ , Zheng BQ, Wang JY et al. New insight into the molecular mechanism of colour differentiation among floral segments in orchids. Commun Biol. 2020;3:89. 32111943 10.1038/s42003-020-0821-8 PMC7048853

[206] Anwar M , Yu W, Yao H et al. *NtMYB3*, an R2R3-MYB from *narcissus*, regulates flavonoid biosynthesis. Int J Mol Sci. 2019;20:5456. 10.3390/ijms20215456 PMC6862390 31683873

[207] Zhao Q , Dixon RA. Transcriptional networks for lignin biosynthesis: more complex than we thought? Trends Plant Sci. 2011;16:227–33. 21227733 10.1016/j.tplants.2010.12.005

[208] Sun Y , Ren S, Ye S et al. Identification and functional characterization of *PtoMYB055* involved in the regulation of the lignin biosynthesis pathway in *Populus tomentosa*. Int J Mol Sci. 2020;21:4857. 10.3390/ijms21144857 PMC7402297 32659969

[209] Jiao B , Zhao X, Lu W et al. The R2R3 MYB transcription factor MYB189 negatively regulates secondary cell wall biosynthesis in Populus. Tree Physiol. 2019;39:1187–200. 30968143 10.1093/treephys/tpz040

[210] Fornalé S , Sonbol FM, Maes T et al. Down-regulation of the maize and *Arabidopsis thaliana* caffeic acid O-methyl-transferase genes by two new maize R2R3-MYB transcription factors. Plant Mol Biol. 2006;62:809–23. 16941210 10.1007/s11103-006-9058-2

[211] Huang J , Guo Y, Sun Q et al. Genome-wide identification of R2R3-MYB transcription factors regulating secondary cell wall thickening in cotton fiber development. Plant Cell Physiol. 2019;60:687–701. 30576529 10.1093/pcp/pcy238

[212] Ye Y , Liu B, Zhao M et al. CEF1/OsMYB103L is involved in GA-mediated regulation of secondary wall biosynthesis in rice. Plant Mol Biol. 2015;89:385–401. 26350403 10.1007/s11103-015-0376-0

[213] Xue C , Yao JL, Xue YS et al. *PbrMYB169* positively regulates lignification of stone cells in pear fruit. J Exp Bot. 2019;70:1801–14. 30715420 10.1093/jxb/erz039

[214] Zhang J , Ge H, Zhang C et al. EjODO1, a MYB transcription factor, regulating lignin biosynthesis in developing loquat (*Eriobotrya japonica*) fruit. Front Plant Sci. 2016a;7:1360–0. 27695460 10.3389/fpls.2016.01360 PMC5025436

[215] Seo MS , Kim JS. Understanding of MYB transcription factors involved in glucosinolate biosynthesis in Brassicaceae. Molecules. 2017;22:1549. 10.3390/molecules22091549 PMC6151624 28906468

[216] Augustine R , Mukhopadhyay A, Bisht NC. Targeted silencing of *BjMYB28* transcription factor gene directs development of low glucosinolate lines in oilseed *Brassica juncea*. Plant Biotechnol J. 2013;11:855–66. 23721233 10.1111/pbi.12078

[217] Araki R , Hasumi A, Sasaki K et al. Novel bioresources for studies of *Brassica oleracea*: identification of a kale MYB transcription factor responsible for glucosinolate production. Plant Biotechnol J. 2013;11:1017–27. 23910994 10.1111/pbi.12095

[218] Liu Y , Zhou X, Yan M et al. Fine mapping and candidate gene analysis of a seed glucosinolate content QTL, qGSL-C2, in rapeseed (*Brassica napus* L.). Theor Appl Genet. 2020;133:479–90. 31832742 10.1007/s00122-019-03479-x

[219] Yin J , Sun L, Li Y et al. Functional identification of *BpMYB21* and *BpMYB61* transcription factors responding to MeJA and SA in birch triterpenoid synthesis. BMC Plant Biol. 2020;20:374–4. 32787836 10.1186/s12870-020-02521-1 PMC7422618

[220] Hao X , Pu Z, Cao G et al. Tanshinone and salvianolic acid biosynthesis are regulated by *SmMYB98* in *Salvia miltiorrhiza* hairy roots. J Adv Res. 2020;23:1–12. 32071787 10.1016/j.jare.2020.01.012 PMC7016019

[221] Liu L , Yang D, Xing B et al. *SmMYB98b* positive regulation to tanshinones in *Salvia miltiorrhiza* Bunge hairy roots. Plant Cell Tissue Organ Cult. 2020;140:459–67.

[222] Liu T , Luo T, Guo X et al. *PgMYB2*, a MeJA-responsive transcription factor, positively regulates the dammarenediol synthase gene expression in *Panax Ginseng*. Int J Mol Sci. 2019;20:2219. 10.3390/ijms20092219 PMC6539309 31064108

[223] Reddy VA , Wang Q, Dhar N et al. Spearmint R2R3−MYB transcription factor MsMYB negatively regulates monoterpene production and suppresses the expression of geranyl diphosphate synthase large subunit (*MsGPPS*. *LSU*). Plant Biotechnol J. 2017;15:1105–19. 28160379 10.1111/pbi.12701 PMC5552485

[224] Soler M , Corocha V, Cassan-Wang H et al. The Eucalyptus grandis R2R3-MYB transcription factor family: evidence for woody growth-related evolution and function. New Phytol. 2015;206:1364–77. 25250741 10.1111/nph.13039

[225] Li X , Xue C, Li J et al. Genome-wide identification, evolution and functional divergence of MYB transcription factors in Chinese white pear (*Pyrus bretschneideri*). Plant Cell Physiol. 2016;57:824–47. 26872835 10.1093/pcp/pcw029

[226] Zhao K , Bartley LE. Comparative genomic analysis of the R2R3 MYB secondary cell wall regulators of *Arabidopsis*, poplar, rice, maize, and switchgrass. BMC Plant Biol. 2014;14:135. 24885077 10.1186/1471-2229-14-135 PMC4057907

[227] Stracke R , Holtgrawe D, Schneider J et al. Genome-wide identification and characterisation of R2R3-MYB genes in sugar beet (*Beta vulgaris*). BMC Plant Biol. 2014;14:17. 25249410 10.1186/s12870-014-0249-8 PMC4180131

[228] Hatlestad GJ , Akhavan NA, Sunnadeniya RM et al. The beet Y locus encodes an anthocyanin MYB-like protein that activates the betalain red pigment pathway. Nat Genet. 2015;47:92. 25436858 10.1038/ng.3163

