# Supplementary material for: Evolution and functional diversification of R2R3-MYB transcription factors in plants
Source: Hortic Res. 2022 Mar 8;9:uhac058. doi: 10.1093/hr/uhac058 (PMC9113232; doi:10.1093/hr/uhac058)
Supplement: Web_Material_uhac058 [file web_material_uhac058.zip › Supplemental Fig. 1.pdf]

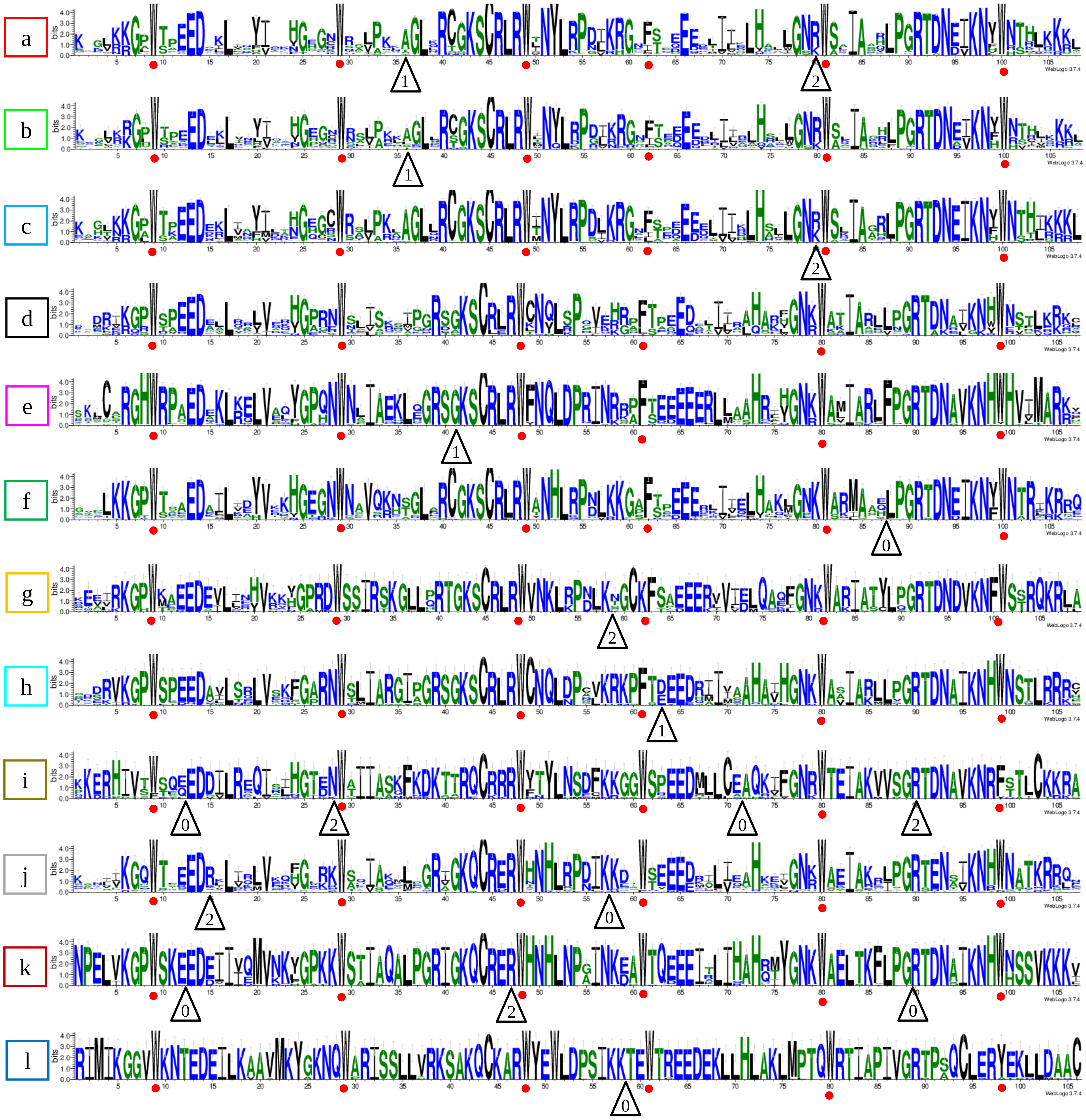

Supplemental Fig. 1 Intron patterns within the MYB domains of *R2R3-MYB* genes in land plants. The sequence logos of the MYB domains of R2R3-MYB proteins from *Physcomitrella patens*, *Selaginella moellendorfii*, *Zea mays*, *Oryza sativa*, *Aquilegia coerulea*, *Solanum lycopersicum*, *Solanum tuberosum*, *Vitis vinifera*, *Arabidopsis thaliana*, *Citrus sinensis*, *Medicago truncatula*, *Populus trichocarpa* and *Brassica napus* were generated by Weblogo 3 (http://weblogo.berkeley.edu/) based on amino acid alignment using MAFFT (https://mafft.cbrc.jp/alignment/server/). The candidate R2R3-MYB sequences were obtained from our previous reports (Du et al., 2015; Li et al., 2020). The bitscore indicates the information content for each position in the sequences. The height of the letter represents the degree of amino acid residue conservation at each position. Red dots indicate conserved Trp (W) residues in the MYB domain.
