# Supplementary material for: Evolution and functional diversification of R2R3-MYB transcription factors in plants
Source: Hortic Res. 2022 Mar 8;9:uhac058. doi: 10.1093/hr/uhac058 (PMC9113232; doi:10.1093/hr/uhac058)
Supplement: Web_Material_uhac058 [file web_material_uhac058.zip › Supplemental Fig.2.pdf]

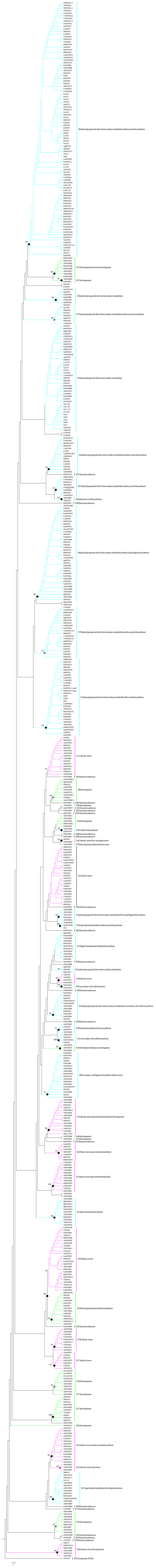

Supplemental Fig. 2 Phylogenetic tree of functionally characterized plant *R2R3-MYB* genes. The tree was constructed by the Maximum likelihood (ML) method using JJT+G model, based on the alignment of the MYB domains of 598 nonredundant R2R3-MYB proteins of the 126 *Arabidopsis R2R3-MYB* genes (Stracke et al., 2001), the representatives of 73 subfamilies in our previous results (Du et al., 2015), and 435 functionally characterized *R2R3-MYB* genes are listed in Supplemental Table 2. The candidate R2R3-MYB proteins are clustered into 79 subfamilies (designated as S1 to S79). The first 25 subfamilies were according to a previous report on *Arabidopsis* R2R3-MYB gene family (Stracke et al., 2001), whereas remaining subfamilies were according to our previous reports in plants (Du et al., 2015; Li et al., 2020). The tree was rooted using S29 (the CDC5-like protein) as the outgroup. The numbers beside the branches represent bootstrap values based on 100 replications. Bootstrap values ≥70% and ≥50% are shown as black and gray dots in the phylogenetic tree, respectively, while those <50% are not shown. The major functions of each subfamily are listed in parentheses for reference, and detailed information about the function of the corresponding subfamilies is provided in Supplemental Table 2.
