# Supplementary material for: Evolution and functional diversification of R2R3-MYB transcription factors in plants
Source: Hortic Res. 2022 Mar 8;9:uhac058. doi: 10.1093/hr/uhac058 (PMC9113232; doi:10.1093/hr/uhac058)
Supplement: Web_Material_uhac058 [file web_material_uhac058.zip › MYB-review-manuscript-revision V2_with track changes.doc]

Title: Evolution and Functional Diversification of R2R3-MYB Transcription Factors in Plants

**Running Title:** R2R3-MYB transcription factor family in plants

**Authors:** Yun Wu^1, 3#^, Jing Wen^2#^, Yiping Xia^3^, Liangsheng Zhang^3*^, Hai Du^2*^

**Affiliations:** ^1^Department of Landscape Architecture, School of Civil Engineering and Architecture, Zhejiang Sci-Tech University, Hangzhou, China；^2^ College of Agronomy and Biotechnology, Southwest University, Chongqing, 400716, China; ^3^Genomics and Genetic Engineering Laboratory of Ornamental Plants, College of Agriculture and Biotechnology, Zhejiang University, Hangzhou, China

^#^ co-first author

**^*^Corresponding author:**

Liangsheng Zhang, E-mail: zls83@zju.edu.cn；Hai Du, E-mail: haidu81@126.com

**E-mail addresses**

Y.W: yunwu@zju.edu.cn; JW: luckywenjing93@126.com; Y.P.X: ypxia@zju.edu.cn; L.S.Z: zls83@zju.edu.cn; H.D: dh20130904@swu.edu.cn/haidu81@126.com.

Abstract: R2R3-MYB genes (*R2R3-MYBs*) form one of the largest transcription factor gene families in the plant kingdom, with substantial structural and functional diversity. However, the evolutionary processes leading to this amazing functional diversity have not been clearly established yet. Recently developed genomic and classical molecular technologies have provided detailed insights into the evolutionary relationships and functions of plant *R2R3-MYBs*. Here, we review recent genome-level and functional analyses of plant *R2R3-MYBs,* with an emphasis on their evolution and functional diversification. In land plants, this gene family underwent a large expansion by whole genome duplications and small-scale duplications. Along with this population explosion, a series of functionally conserved or lineage-specific subfamilies/groups arose with roles in three major plant-specific biological processes: development and cell differentiation, specialized metabolism, and biotic and abiotic stresses. The rapid expansion and functional diversification of plant *R2R3-MYBs* are highly consistent with the increasing complexity of angiosperms. In particular, recently derived *R2R3-MYBs* with three highly homologous intron patterns (a, b, and c) are disproportionately related to specialized metabolism and have become the predominant subfamilies in land plant genomes. The evolution of plant *R2R3-MYBs* is an active area of research, and further studies are expected to improve our understanding of the evolution and functional diversification of this gene family.

# **Introduction**

The V-myb avian myeloblastosis viral oncogene homolog (MYB) genes encode a group of pan-eukaryotic transcription factors (TFs) characterized by a highly conserved N-terminal DNA-binding domain repeat (R) plus a variable C-terminal regulatory region^1-6^. MYB genes constitute the second largest TF superfamily in flowering plants^7^, accounting for ~13% of the 1,500 TFs in the model plant *Arabidopsis thaliana*^8^. Conversely, only a few MYB genes are present in unikonts^9,10^; for example, only three have been reported in the *Homo sapiens* genome, the human c-MYB proto-oncogene product (c-MYB) and two related vertebrate MYB factors^11,12^. Each MYB repeat contains approximately 50 amino acid residues forming three α-helices and special DNA motifs, including MYB binding site I (MBSI), MBSII, or MBSIIG^12-15^, with some exceptions^16^. According to sequence similarity, MYB domain repeats are named R1, R2, or R3 type^5,17^. Typically, plant MYB superfamily members include 1–4 imperfect MYB repeat(s) and are therefore subdivided into four families: 1R-MYB (consisting of one or two separated repeats), 2R-MYB (R2R3-MYB, consisting of two adjacent repeats), 3R-MYB (consisting of three adjacent repeats), and 4R-MYB (consisting of four adjacent repeats)^5^. The R2R3-MYB family expanded substantially in the plant lineage and is the predominant family^6,18,19^.

Since the first plant R2R3-MYB gene, *COLORED1 (C1)*, was identified in *Zea mays* and was demonstrated to be essential for anthocyanin biosynthesis in aleurone tissues^20^, a tremendous number of R2R3-MYB genes (R2R3-MYBs) have been identified. In the past decade, based on substantial genome sequence data, the whole R2R3-MYB gene family has been comprehensively identified in numerous plant genomes (Supplemental Table 1), with counts ranging from dozens to hundreds, e.g., 126 genes in *A. thaliana*^9^, 192 in *Populus*^21^, 244 in soybean^22^, and 406 in *Gossypium hirsutum*^23^. A comparative phylogenetic analysis of R2R3-MYBs revealed considerable diversiﬁcation and conservation of this gene family in plants^10^. Concomitantly, the functions and characteristics of R2R3-MYB proteins have been studied extensively (Supplemental Table 2), including roles in various plant-specific processes, such as responses to ambient stimuli^24-29^, specialized metabolism^5,20,30-34^, development^35-38^, and cell differentiation^39-41^. Given their important roles in plants, the separate biological processes regulated by R2R3-MYBs have been reviewed in detail elsewhere recently^42-47^.

A number of studies have proven that this gene family underwent a large expansion with functional diversification in land plants^6,9,10,18,19,21,49^. This may have contributed to the origin and diversity of this kind of plants, as it conferred them the ability to adapt from aquatic to terrestrial environments during evolution^50^. To capture the most significant developments in this area, this review focuses on (a) the identification and classification of the R2R3-MYB gene family in plants, (b) the characterization of conserved and diverse functions in different species and biological processes, (c) and the application of comparative genomics to clarify the R2R3-MYB family expansion and functional diversification. Finally, we discuss new research directions to improve our understanding of R2R3-MYB evolution and functions.

# **Genome-wide characteristics and evolution of the R2R3-MYB gene family in plants**

## *Identification and classification of R2R3-MYBs*

Since it was initially identified in 1982^1^, the *R2R3-MYB* family has been an important area of research. In the past 20 years, an increasing number of sequenced plant genomes have enabled integrative genome-wide overviews of this gene family in the plant kingdom. To date, the R2R3-MYB gene family has been evaluated in about 74 plant species, ranging from aquatic plants (such as *Mesostigma viride*^51^) to angiosperms (e.g., *A. thaliana*^9^, *Z. mays*^49^, *P. trichocarpa*^21^, *Gossypium* spp.^23^, and *Solanum tuberosum*^52^) (Fig. 1 and Supplemental Table 1), and a pipeline for the automatic identification of MYBs has been created, which could make genome-wide (or transcriptome assemblies) investigations consistent^53^. These studies have revealed that *R2R3-MYBs* are widely distributed in the plant kingdom, with a sharp increase in number from aquatic to angiosperm plants, forming one of the largest and most diverse TF gene families (ranging from one to hundreds of members) in land plants, especially in angiosperms (Fig. 1).

The gene family clearly underwent functional diversification to form different subfamilies/groups during evolution. Accordingly, there are many classification schemes for plant R2R3-MYBs, and the differences primarily reflect the existence of lineage- or species-specific “orphan” genes^54,55^, differences in sampling coverage, and phylogenetic analysis methods^6^. On the whole, three landmark classification systems have been developed and improved. First, the canonical classification and nomenclature of this gene family were initially established in *A. thaliana*^9^*,* including 25 groups based on the MYB domain and the amino acid motifs in the C-terminal regions^5^. Thereafter, many studies have applied this system, with varying degrees of modification^22,49,56-58^. Notably, in *A. thaliana*^9^, around 30 of the 126 (~23.81%) *R2R3-MYBs* were not assigned to any subfamily/subgroup in this classification owing to the lack of different representative species and the limited gene number (generally only one per species), resulting in scattered studies with relatively small sample sizes. Second, we performed a systematic comparative analysis of 50 major eukaryotic lineages with a total of 1548 *R2R3-MYBs*^10^. As expected, we not only confirmed the 25 well-defined subfamilies of the R2R3-MYB gene family in *A. thaliana*^9^ but also defined many new species- or lineage-specific subfamilies in major eukaryotic lineages that were previously neglected^21^; ultimately, we classified the gene family into as many as 73 subfamilies supported by highly conserved gene structures and motif compositions^10^. The validity of this classification was further supported by our recent study of Brassicaceae^18^. In addition, a more systematic classification with a high resolution based on 87 species and 4312 sequences has recently been released, covering the major lineages of Archaeplastida. In this study, ten clades were designated as land plant R2R3-MYB subfamilies by applying a progressive phylogenetic analysis strategy aimed at eliminating the imbalance in resolution and species sampling^6^. We speculated that there may be more subfamilies specific to individual species or lineages, as there are still many “orphan” genes in the phylogenetic analyses. Third, the genome sequences for *Marchantia polymorpha*^59^ and *Mesostigma viride*^51^ have enabled a macroscopic classification regime resulting in three clades of *R2R3-MYBs* corresponding to their biological functions. Each clade is correlated with different functions associated with essential developmental processes, from the basic life cycle to specialized metabolism and organismal complexity, corresponding to a very recent classification in 2020^60^. In this study, genes encoding R2R3-MYBs from Rhodophytes, Glaucophytes, Chromista, Chlorophytes, Charophytes, and Embryophytes were divided into three major clades (I, II, and III) (named subgroups in the original paper). Subgroup I is the most ancient group and includes members from all plant lineages, while subgroup III has become predominant in land plants^60^. The former two classification schemes share overlap and are based on domain conservation, while this final classification system provides a macroscopic perspective based on broad evolutionary patterns.

Notably, a uniform nomenclature within the classification of this gene family in plants is still lacking. Currently, there is almost no dispute at the family (R2R3-MYB) and superfamily (MYB) levels, except for a few studies that refer to *R2R3-MYBs* as a subfamily or subgroup^50,61^. However, the nomenclature after R2R3-MYB is inconsistent, e.g., ~7% of studies refer to groups^62,63^, ~31% refer to subfamilies^10,64^, and ~54% refer to subgroups^23,65^, with clade or cluster used occasionally^66,67^. Subfamilies and subgroups are the most common subsequent appellations after family. Given that subfamily is used after the family level in many other TF gene families, e.g., basic helix-loop-helix proteins (bHLHs)^68^, MADS-Box^69^, and GRAS^70^, we propose an improved nomenclature system from superfamily (MYB) and family (R2R3-MYB) to subfamily. “Subgroup” used by Chang and colleagues should be revised to evolutionary clade/group^60^. To avoid confusion, in the following sections, we uniformly refer to the lower classification within the gene family as subfamilies (abbreviated as S1, S2, etc.).

## *Structural conservation of R2R3-MYBs*

The R2R3-MYB protein consists of two major functional parts: a DNA-binding domain (MYB-domain) located at the N-terminus and regulatory regions (non-MYB region) at the C-terminus. The MYB-domain is a signature, highly conserved feature in the gene family, while the non-MYB regions have diverged among plant species (Fig. 2a). Additionally, the intron patterns in the MYB domains of the *R2R3-MYBs* are highly conserved within each subfamily across land plants^6,9,10,22,49^. In most cases, the MYB domain has one or two conserved intron insertion sites, with rare intron-less and multi-intron genes^6,10^. Although the number of intron patterns differs slightly among taxonomic groups, there is clearly high structural conservation of MYB domains across land plants. In our previous study, we identified 12 highly conserved intron patterns across the plant kingdom^10^ that may have arisen early in the transition from aquatic to terrestrial plants based on conserved intron numbers, insertion positions, and intron phases (Supplemental Fig. 1). The majority (~70%) of the tested *R2R3-MYBs* share three types (patterns “a”, “b” and “c”) of the 12 intron patterns (patterns “a” to “l”)^10^, suggesting biased expansion during evolution. A recent study has shown that some charophyte *R2R3-MYBs* exhibit intro–exon structures identical to those of their corresponding land homologs in the same subfamily, while some showed clear differences, indicating a difference among ancestral genes^6^.

R2R3-MYB proteins can act as transcriptional activators as well as repressors^42^. Most are activators that bind to cis-elements in gene promoters to recruit transcriptional machinery and stimulate gene expression^43^. For instance, many R2R3-MYBs have a bHLH-interacting motif ‘(D/E)Lx2(R/K)x3Lx6Lx3R)’ in the R3 MYB domain (Fig. 2a), enabling physical interactions with the bHLH protein to form the MYB-bHLH-WDR (MBW) transcriptional complex^48^. Notably, although the conservation of the non-MYB region is much lower than that of the MYB domain, the C-terminal regions of closely related members within each subfamily or close subfamilies in an evolutionary clade generally share short conserved protein motifs (some called short linear motifs, SLiMs), such as EAR (ethylene-responsive element binding, ‘pdLNL(D/E)L’ or ‘DLNxxP’ in S4)^71^, ‘(R/K)PRPRx(F/L)’ in S6, SID (sensitive to ABA and Drought 2 protein interact motif), and TLLLFR^72^ (Fig. 2a), indicating their common origin, functional similarities, and structural conservation^9^. Recently, Millard et al.^54^ identified that the interactions between MYB TFs from S12 and bHLH were mediated by one of the conserved motifs ([L/F]LN[K/R]VA) located in the center of the non-MYB region. Two novel SLiMs were uncovered by Rodrigues et al.^73^ in the disordered C-terminal region with roles in protein-protein interaction. For more detailed information, see the recent review by LaFountain and Yuan^47^ and Chen et al.^35^. MYB activators and repressors often operate in a hierarchy^74^. Furthermore, many subfamily- and/or branch-specific motifs have been detected within R2R3-MYBs. For instance, based on a large number of species, up to 102 highly conserved motifs have been identified in non-MYB regions^10^. Interestingly, non-MYB regions across the entire *A. thaliana* R2R3-MYB gene family contain extensive intrinsically disordered regions (IDRs) with special post-translational modification sites, binding sites for physical interactions, and activation domains. These features may explain the substantial functional diversity and the structure–function relationships in plant R2R3-MYBs^54^. However, owing to the high divergence in non-MYB regions, a systematic analysis of motif(s) and/or IDRs covering broad and representative plant lineages is still lacking. Moreover, further experimental evidence is needed, including for IDRs.

# **Functional conservation and diversification of *R2R3-MYBs* in land plants**

The biological functions of *R2R3-MYBs* have been verified in an increasing number of non-model crops with high economical value, especially in the last decade. More than 500 *R2R3-MYBs* have been functionally characterized in around 130 plant species (Fig. 2b and Supplemental Table 2), and their roles in many biological processes have been reported^5,27-29,32-34,38,41,75-77^. Notably, research progress related to R2R3-MYB gene function is mainly based on studies on the model plant *A. thaliana*; approximately 80% of its 126 R2R3-MYBs have been experimentally characterized (Fig. 2c). Overall, the functions of *R2R3-MYBs* could be classified into three major processes: development and cell differentiation, specialized metabolism (especially the phenylpropanoid biosynthesis pathway), and stress responses (biotic and abiotic stresses).

## *R2R3-MYBs are required for developmental and cell differentiation processes*

Plant *R2R3-MYBs* are involved in the regulation of many important aspects of plant growth and development (Supplemental Table 2), e.g., trichome initiation and branching^78,79^, cuticle development^80^, and regulation of different organs, including the flower, seed^81,82^, shoot^41,83,84^, and root^85,86^. In general, genes belonging to the S9 (e.g., *AtMYB16*), S15 (e.g., *AtMYB66*), S18 (e.g., *AtMYB33*), S25 (e.g., *AtMYB64*), and S27 (e.g., *AtMYB91*) subfamilies^9^ are exclusively associated with the above-mentioned biological functions (Supplemental Table 2). For instance, genes in S9 are involved in cuticle formation and trichome branching in *A. thaliana* (*AtMYB16* and *AtMYB106*)^80^ and *M. polymorpha* (*MpSBG9*)^87^, conical epidermal cell outgrowth in *Antirrhinum majus* (*AmMYBML1*)^88^, photosynthesis and growth in *Betula platyphylla* (*BpMYB106*)^89^, and fiber development in cotton (*GhMML3*)^90^. The genes in S18 mainly regulate flower organ development, such as stamen (*AtMYB33*, *AtMYB65*, and *BcMF28*)^91,92^, anther (*OsGAMYB*) ^93^, and pollen tube-synergid interactions (*AtMYB97*, *AtMYB101*, and *AtMYB120*)^94^. Interestingly, the above processes are usually mediated by hormone and/or hormone signaling, e.g., JA and GA. *R2R3-MYBs* are also involved in plant reproductive growth, e.g., members of S25^95^ (Supplemental Table 2). More recently, *MpFGMYB*, a member of S25, has been identified as a critical factor in sexual dimorphism determination in *M. polymorpha*^96^, and similar results regarding spore and sexual organ development were obtained in *Selaginella moellendorffii* (*SmGAMB*) and *Physcomitrella patens* (*PpGAMYB1* and *PpGAMYB2*)^97^. Members of S27 contribute to the regulation of vegetative organ formation, including leaf patterning and development (*NbPHAN* and *PHANTASTICA*) ^98,99^, stomata cell differentiation and formation^16^, and axillary meristem formation (*ZmRS2* and *SlPHAN*)^100-102^ (Supplemental Table 2). S14 also functions in meristem initiation as well as root formation (*CRY1*)^103,104^ (Supplemental Table 2). A good example in this subfamily is the *MpGCAM1* gene, which precisely controls gemma cup formation in liverwort^105^. Another example of genes regulating cell growth during gametophytic development are *MpPp1* and *MpPp2* in *P. patens*^106^. Furthermore, some subfamilies have species-specific functions. For instance, *AtMYB21*, *AtMYB24*, and *AtMYB57* in S19 have key roles in stamen development^107,108^. However, their homologs in almost all other species studied, including *Fressia* hybrids^109,110^, *Gentian tutea*^111^, *Ficus carica*^112^, *Malus domestica*^113^, and *Petunia* *hybrida*^114^, regulate specialized metabolism (Supplemental Table 2). Further research should focus on S16^115^, S24^116^, S28^117^, S31^118^, S33^119^, S36^120^, and S37^121,122^, for which experimental data are rather limited (Supplemental Table 2).

In summary, *R2R3-MYBs* are a class of important TFs necessary for the basic life cycle in plants. Therefore, there is a need for more extensive field studies of *R2R3-MYBs* in the future to determine their functions in basic developmental processes.

## *R2R3-MYBs respond to various environmental stresses*

Over the past few decades, *R2R3-MYBs* have emerged as key regulators of responses to diverse abiotic stresses, such as drought, temperature, salinity, and phosphate starvation^28,123-127^ (Supplemental Table 2); see previous reviews^75,128,129^. Representative subfamilies are S1, S2, S11, S17, S20, S22, and S38. In *A. thaliana*, members of S1 (*AtMYB30*, *AtMYB60,* and *AtMYB96*) are involved in drought, heat, salt, and excess-light stresses^126,130-137^; members of S2 (*AtMYB13*, *AtMYB14*, and *AtMYB15*) act as positive regulators of drought, wounding, and freezing tolerance^138-141^; *AtMYB41* and *AtMYB74* in S11 are involved in osmotic and/or salt stress^142,143^; members of S20 (*AtMYB2*, *AtMYB62*, and *AtMYB108*) are responsive to a wide range of environmental stresses^144-146^; similar roles have been observed in S22 members (*AtMYB44*, *AtMYB73* and *AtMYB77*)^26,147,148^. *AtMYB48* in S38 is involved in potassium stress possibly via alternative splicing, which modulates DNA-binding motifs^149^. Some genes belonging to S3^150^, S4^125^, S8^151^, and S23^152^ also show stress-induced functionality in *A. thaliana*. In addition to the *R2R3-MYBs* in *A. thaliana*, around 70 genes identified in 26 species have also been found to be involved in distinctive abiotic stresses (Supplemental Table 2). As in *A. thaliana*, abundant proteins are distributed in S2^153-155^, S11^156-158^, S20^159-161^, and S22^162-165^. However, distinct differences have been noted. Some groups have been confirmed only in *A. thaliana* (e.g., *AtMYB1* from S23a, and *AtMYB72* from S3)^150,1152^ or solely in non-model species, such as S44, S4, S17, S28, S43, S7, S39, and S79^123,127,166-178^. Taken together, these results suggest that *R2R3-MYBs* control multiple abiotic stresses and act as major regulators. However, more research is needed to improve our understanding of the functional divergence and conservation of this gene family within plants, which could provide a theoretical basis for the development of strategies to resolve the adverse effects of abiotic stresses, which are exacerbated by climate change.

Many R2R3-MYBs contribute to the biotic stress response, and at least 13 *R2R3-MYBs* have been reported to date (Supplemental Table 2). Interestingly, eight of these genes belong to S2. *OsMYB30* directly up-regulates *OsPAL6* and *OsPAL8* to enhance resistance to the brown planthopper in rice^29^. The overexpression of *CmMYB15* in chrysanthemum confers resistance to aphids by regulating lignin biosynthesis^179^. *GmMYB29A2* regulates soybean resistance to *Phytophthora sojae*^180^. *SlHM1* and *SlMYB52* alter trichome density to confer spider mite tolerance in tomato via auxin signaling^118^. *VdMYB1* in *Vitis davidii*^181^ and *TaRIM1* in wheat regulate pathogen defense^182^. Other examples include *AtMYB30* (S1)^183^, *AtMYB96* (S1)^184^, *AtMYB102* (S11)^185^, and *AtMYB72* (S3)^186,187^ in *A. thaliana* and *OsJAmyb* (S39) in *Oryza sativa*^188^.

In summary, *R2R3-MYBs* could improve plant responses to multiple abiotic and biotic stresses in agriculture and could be targets of advanced technologies, such as CRISPR technology, to improve crop resistance to environmental stimuli aimed at maintaining the food supply.

## *Key roles of R2R3-MYBs in metabolite biosynthesis*

The principal roles of plant *R2R3-MYBs* (~70%) reported to date are the regulation of plant specialized metabolism, including the benzenoid, phenylpropanoid, terpenoid, and glucosinolate (GSL) pathways (Supplemental Table 2). Nearly half of the functionally characterized *A. thaliana R2R3-MYBs* are related to core and specialized metabolism reactions (Fig. 3 and Supplemental Table 2)^5,9^. Notably, the majority of these *R2R3-MYBs* are involved in the transcriptional regulation of the phenylpropanoid biosynthesis pathway, giving rise to a class of phenylpropanoid-derived compounds, such as flavonoids (proanthocyanidins, anthocyanins, flavones, flavonols, isoflavonoids, and phlobaphenes**)**, lignins, and other general phenylpropanoids (Fig. 3a), either as activators or repressors^43,77,189^.

Flavonoids are a vast group of plant specialized metabolites bearing a common diphenylpropane (C6-C3-C6) backbone^192^, with important functions as pigments and/or light protectants. Typical subfamilies in the R2R3-MYB gene family that are involved in the regulation of flavonoid biosynthesis are S6, S5, S4, S7, S44, and S79 (Supplemental Table 2). Beyond *A. thaliana*, there is substantial emerging evidence for the roles of *R2R3-MYBs* in the transcriptional regulation of flavonoid metabolism in various plants, including important food crops (41), horticultural crops (fruits, 77; ornamentals, 61; vegetables, 37), economical woody crops (27), and edible and medicinal plants (30). With respect to edible crops, including fruits and vegetables, research has mainly focused on the substances determining the quality and nutritional properties, such as anthocyanins and flavonols. Nevertheless, flower colors are key traits for flower crops^42^. For instance, potato *StMYB44* has recently been identified as a repressor of anthocyanin biosynthesis in tuber flesh under high temperatures^193^, and whole-genome resequencing-based QTL-seq indicated that *AhTc1* controls the peanut purple testa color^194^. Additionally, the overexpression of *MdMYB24L* resulted in higher anthocyanin contents in the transgenic apple ‘Orin’ calli^113^. Similar studies have been performed in other fruit trees, including *PaMYB10* in apricot^195^, *AcMYB123* in kiwifruit^32^, *LcMYB5* in litchi^196^, and *FcMYB123* in fig^112^. In *M. polymorpha*, *MpMYB14* and *MpMYB02* regulate anthocyanin accumulation^197^. Moreover, different types of flavonoid regulation are broadly observed. For instance, the regulatory roles of *PhMYB15* and *PpMYBF1* in flavonol biosynthesis in peach fruit^198^, *FhMYB21L2* in flavonol biosynthesis in *Freesia*^109^, *CsPH4* in proanthocyanidin biosynthesis in citrus^199^, *SlMYB72* in carotenoid biosynthesis in tomato^200^, *CaMYB108* in capsaicinoid biosynthesis in pepper^201^, *AgMYB1* in apigenin biosynthesis in celery^137^, *HaMYB111* in floral ultraviolet patterning in sunflower^202^, and *SmMYB2* in phenolic acid biosynthesis^203^ have been reported. Of note, apart from model flowers, *viz.*, *Petunia* and *Antirrhinum*, a number of flowering bulbs have recently been studied, including *Freesia*^204,205^, *Cattleya*^206^, and *Narcissus*^207^.

Lignin is another major end-product of the phenylpropanoid pathway and a key component of secondary cell walls in wood; it is polymerized from phenylpropanoid-derived monolignols^208^. The role of *R2R3-MYBs* in the lignin biosynthesis pathway is a major focus of research. As wood is widely used in pulp, papermaking, and biofuels and wood quality is largely determined by lignin synthesis, most studies of the regulatory effects of R2R3-MYB on lignin synthesis are based on woody plants. For example, the overexpression of *PtoMYB055* upregulates the expression of lignin biosynthetic genes in transgenic poplar plants, resulting in an increase in the thickness of the secondary cell wall (SCW)^209^. In contrast, *PtMYB189* acts as a repressor to regulate SCW biosynthesis in poplar^210^. Likewise, the positive or negative regulation of SCW by R2R3-MYBs has also been reported in *Z. mays*^211^, *G. hirsutum*^212^, *O. sativa*^213^, *Pyrus* × *bretschneideri*^214^, and *Eriobotrya japonica*^215^; however, additional studies are still needed.

In addition to phenylpropanoid metabolism, which has been investigated extensively in plants, other representative specialized metabolic compounds have been studied, such as GSL and terpenoid pathways (Fig. 3b–c). GSLs have well-established anticarcinogenic and antioxidative effects in humans^216^. Interestingly, consistent with the distribution of GSLs, members of S12 are widely distributed in species belonging to the family Brassicaceae and are predominantly related to GSL biosynthesis in this family (Supplemental Table 2). It has been summarized that indolic GSL synthesis is mainly regulated by *AtMYB34*, *AtMYB51*, and *AtMYB122*, while aliphatic GSL synthesis is regulated by At*MYB28*, At*MYB29*, and *AtMYB76*^191^. For example, silencing *BjMYB28* homologs reduces the seed GSL content in *Brassica juncea*^2017^, *BoMYB29* increases the methysulphinyl GSL content in *Brassica oleracea* var. *acephala*^218^, and *BnMYB28.1* was identified by a QTL analysis using near-isogenic lines^219^. These studies provide good examples of the adaptive evolution of the R2R3-MYB gene family in land plants, especially in angiosperm diversification. Some plants can produce and emit fragrant molecules, such as benzenoids and terpenoids, especially flowers, and these processes are strictly regulated at the transcriptional level by *R2R3-MYBs*. For instance, two *R2R3-MYBs*, *FhMYB21L1* and *FhMYB21L2*, are expressed synchronously with *FhTPS1* and could activate its expression to impact monoterpene synthase synthesis when overexpressed^110^. Additionally, *BpMYB21* and *BpMYB61* in *Betula platyphylla* are involved in triterpenoid synthesis^220^. In several medicinal plants, including *Salvia miltiorrhiza*^221,222^, *Panax ginseng*^223^, and *Mentha spicata*^224^, *R2R3-MYBs* regulate terpenoid compounds.

The research summarized above clearly demonstrates that plant *R2R3-MYBs,* which are related to plant specialized metabolism, are mainly involved in the phenylpropanoid biosynthesis pathway and each subfamily member has key roles in distinct processes, such as proanthocyanidin and lignin biosynthesis. Accordingly, this gene family likely contributed to functional evolution in land plants.

# **The origin and expansion of *R2R3-MYBs* in land plants are associated with functionality**

Gene duplication is a prominent event in plant genome evolution and contributes to the establishment of multi-gene families. Genes can be duplicated via various mechanisms, such as whole genome duplication (WGD), chromosomal segmental duplication, tandem duplication, and retrotransposition. Based on systematic analyses of many land plants, we have previously found that WGD/polyploidy events and small-scale duplication events (e.g., segmental duplication) accounted for the large expansion of the R2R3-MYB gene family^10,18,22^. Similar results have been obtained in studies of broad plant lineages^21,56,225,216^. It is well known that WGD events are a driving force in angiosperm diversification. Coincidentally, the tremendous expansion of *R2R3-MYBs* in land plants is consistent with WGD in angiosperms, indicating an important role for the expansion of *R2R3-MYBs* in the increased complexity of angiosperms. Small-scale duplications (e.g., tandem/segmental duplication events) also contributed to the rapid expansion and large size of this gene family in land plants as well as the evolution of novel gene functions^10,18,21,56,225,226^.

*R2R3-MYBs* underwent a rapid expansion along with the evolution of plants (i.e., from algae to land plants), resulting in a gradual increase in number along with an increase in organismal complexity. Accordingly, the number of genes in this family in land plants is large, showing a huge expansion after the divergence of angiosperms from vascular plants^10,60^. For example, only eight *R2R3-MYBs* were detected in a single-celled chlorophyte, *Chlamydomonas reinhardtii*, while up to 429 genes were observed in the allotetraploid *Brassica napus*^18^ (Fig. 1)*.* Based on the analyses of 50 eukaryotes^10^, the R2R3-MYB gene family was classified into 73 subfamilies, most of which were newly defined (S26-S73) with the exception of the first 25 subfamilies in *Arabidopsis*^9^. Based on the distribution of subfamily members across the 27 plant genomes investigated in this study^10^, it was speculated that a few subfamilies (S18, S21, S22, S26, and S27) were established since the origin of land plant *R2R3-MYBs*, which might be the ancestors of land plant *R2R3-MYBs*, while the major subfamilies formed a monophyletic clade in land plants^10^. In summary, plant *R2R3-MYBs* experienced three major expansion events: early in the origin of land plants from Chlorophyta, after the divergence of spermatophytes from vascular plants, and in the common ancestor of angiosperms predating the divergence of monocot/eudicots, forming the majority of *R2R3-MYB* subfamilies^10,60^. Notably, members of this gene family exhibit an uneven phylogenetic distribution; the expansion was significantly biased toward subfamilies possessing three highly homologous intron patterns (“a,” “b,” and “c”) during evolution, resulting in an enormous expansion in the number of *R2R3-MYBs* in the plant lineage^10,18^ (Fig. 4a and Supplemental Fig. 1). For instance, in the 12 land plants investigated in our previous study^10^, the *2R-MYBs* containing patterns ‘a–c’ generally accounted for ~66‒81% of this gene family in 10 angiosperms, including *Z. mays* (66%), *Arabidopsis* (73%), and *V. vinifera* (81%), whereas the corresponding percentage was 59% and 20% in *P. patens* and *S. moellendorffii*, respectively^10^ (Fig. 4a)*.* A similar situation (73%) was found in *B. napus*^18^. Accordingly, up to 43 subfamilies have intron pattern ‘a’, and 12 and 19 subfamilies have intron patterns ‘a’ and ‘b’, respectively, which were generally integrated into the subfamilies with pattern ‘a’^10^ (Fig. 4b and Supplemental Fig. 2), and the novel subfamilies were commonly derived from these three categories^10,18^. In contrast, the numbers of genes with the last nine intron patterns (“d–l”) are relatively conserved in land plants^10,18^(Fig. 4a). The genes with the last nine intron patterns (“d–l”) are much fewer, generally accounting for less than 30% of this gene family in most angiosperms^10^. Except patterns ‘d’ and ‘j’, which are shared by 7 and 2 subfamilies, respectively, each of the last 7 patterns are shared by only one given subfamily (e.g., pattern ‘e’ in S21, and ‘f’ in S19)^10^ (Fig. 4b and Supplemental Fig. 2). Thus, the number of genes with patterns “d–l” is relatively conserved in land plants^10,18^.

As discussed above, plant *R2R3-MYBs* are crucial for the regulation of many plant-specific processes related to metabolism (22 subfamilies), development (16 subfamilies), and biotic and abiotic stress processes (14 subfamilies) (Fig. 4b and Supplemental Fig. 2). Notably, the expansion of the gene family is closely accompanied by increases in the functional diversity of *R2R3-MYBs* in the plant kingdom. Interestingly, older subfamilies are generally involved in development and/or stress-related processes, while the majority of new/derived subfamilies in angiosperms are related to metabolism (Fig. 4b and Supplemental Fig. 2), suggesting a bias in functional diversification toward specialized metabolism. In particular, the evolution of *R2R3-MYBs* in plants is related to a specific expansion giving rise to species- or lineage-specific subfamilies^225^. For example, 5 of 43 *B. napus* subfamilies are Brassicaceae-specific^18^. Several of the 43 pineapple subfamilies, such as C2, C8, and C22, are likely to be species- or lineage-specific^64^. Similarly, three dicot-specific and six grass-specific subfamilies have been observed based on a comparative genomic analysis of *R2R3-MYBs* in *Arabidopsis*, poplar, rice, maize, and switchgrass^227^. Although the functions of most species- or lineage-specific subfamilies are unknown, the distributions suggest that they have lineage-specific functions. For instance, members of the Brassicaceae-specific S12 (e.g., *AtMYB28/HAG1*, *AtMYB29/HAG3*, *AtMYB34*, *AtMYB51/HIG1*, *AtMYB76/HAG2,* and *AtMYB122* in *A. thaliana*) are regulators of Brassicaceae-specific GSL biosynthesis^5^ (Supplemental Table 2 and Supplemental Fig. 2). Three expanded subfamilies in *E. grandis*, including a significantly higher number in woody perennial species (*E. grandis, V. vinifera,* and *P. trichocarpa*), and five subfamilies preferentially found in woody plants are potentially involved in cambium-derived woody growth^225^. Several sugar beet MYBs, with an atypical amino acid composition in the R3 domain, are species-specific regulators of the betalain red pigment pathway^228,229^. Together, these results indicate an obvious expansion and functional trend toward specialized metabolism in this gene family during angiosperm evolution.

# **Concluding remarks and prospects**

It is known that *R2R3-MYBs* are ubiquitous in eukaryotes but not plant-specific, and the number of this type of MYB genes is generally higher in plants (especially in angiosperms) than in other eukaryotes^10^, constituting one of the largest TF gene families in plant genomes. Giving the increasing number of sequenced plant genomes, the *R2R3-MYB* gene family in the plant kingdom has been systematically identified and analyzed in about 74 species at a genome-wide level (Fig. 1). These data have substantially improved our understanding of its distribution, origin, classification, expansion, and evolution mechanism in plants.

As sessile organisms, plants have to adapt to constantly changing environments during evolution. Through time, plants have differentiated into highly complex organisms and developed a large group of specialized metabolites with beneficial functions for survival and adaptation. *R2R3-MYBs* underwent a rapid expansion during plant evolution via WGD and small-scale duplications. The drastic expansion/duplication events played a key role in generating diversity, including the origin of many conserved subfamilies and even lineage-specific subfamilies with specific biological functions (e.g., S12 in GSL biosynthesis), especially functions related to specialized metabolism. About 70% of functionally characterized *R2R3-MYBs* and most subfamilies are related to specialized metabolism processes focused on phenylpropanoid-derived secondary metabolites (Fig. 4b and Supplemental Table 2). The rapid expansion and diversification of *R2R3-MYBs* are an ongoing process throughout the evolution of angiosperms. It is not surprising that this large gene family has contributed to the evolution of physiological or developmental processes specific to plants. The extension of *R2R3-MYBs* in land plants may underpin the emergence of tremendous plant diversity and provide new functional characteristics related to protection against stress and changing environmental conditions. Thus, uncovering the genomic and molecular basis underlying the origin and evolution of such functional characteristics is a major research goal.

Owing to the availability of resources (e.g., genomic data, genetic populations, and experimental tools) for gene function analyses, a large portion of the *Arabidopsis R2R3-MYB* gene family has been functionally characterized (about 100 of 126 genes). However, our current knowledge of the functions of plant *R2R3-MYBs* is mainly based on the model plant *Arabidopsis*, while studies of non-traditional models (such as crops) are in an early stage. More information about the functions of *R2R3-MYBs* in a broader array of plant taxa will undoubtedly improve our understanding of the mechanisms underlying their evolution and functional diversification. Newly developed genomic technologies (e.g., the third-generation long-read sequencing technology) combined with traditional and novel molecular technologies (e.g., genome editing tools) offer excellent opportunities for further improving our understanding of the functions and functional diversification of *R2R3-MYBs*.

# **Acknowledgments**

This research was funded by the National Natural Science Foundation of China (Grant No. 32072094 and 32002071), the Lishui Research Fund (Grant No. 2020zdhz03), and a start-up fund from Zhejiang Sci-Tech University (Grant No. 21052103-Y).

# **Conflict of interests**

The authors declare that they have no conflict of interest.

# **Author contributions**

H.D., L.Z., and Y.X. conceived the research. Y.W., J.W., and H.D. analyzed the data and wrote the manuscript. Y.W., L.Z., and H.D. discussed and improved the review. All authors read and approved the final manuscript.

**References**

1. Klempnauer, K. H., Gonda, T. J. & Michael Bishop, J. Nucleotide sequence of the retroviral leukemia gene v-MYB and its cellular progenitor c-MYB: The architecture of a transduced oncogene. *Cell* **31**, 453-463 (1982).

2. Lipsick, J. S. One billion years of Myb. *Oncogene* **13**, 223-235 (1996).

3. Rosinski, J. A. & Atchley, W. R. Molecular evolution of the MYB family of transcription factors: evidence for polyphyletic origin. *J. Mol. Evol.* **46**, 74-83 (1998).

4. Kranz, H., Scholz, K. & Weisshaar, B. c-MYB oncogene-like genes encoding three MYB repeats occur in all major plant lineages. *Plant J.* **21**, 231-235 (2000).

5. Dubos, C. *et al*. MYB transcription factors in *Arabidopsis*. *Trends Plant Sci.* **15**, 573-581 (2010).

6. Jiang, C. K. & Rao, G. Y. Insights into the diversification and evolution of R2R3-MYB transcription factors in plants. *Plant Physiol.* **183**, 637-655 (2020).

7. Jin, J., Zhang, H., Kong, L., Gao, G. & Luo, J. PlantTFDB 3.0: a portal for the functional and evolutionary study of plant transcription factors. *Nucleic Acids Res.* **42**, D1182-D1187 (2014).

8. Riechmann, J. L. *et al*. *Arabidopsis* transcription factors: genome-wide comparative analysis among eukaryotes. *Science* **290**,2105-2110 (2000).

9. Stracke, R., Werber, M. & Weisshaar, B. The R2R3-MYB gene family in *Arabidopsis thaliana*. *Curr. Opin. Plant Biol.* **4**, 447-456 (2001).

10. Du, H. *et al*. The evolutionary history of R2R3-MYB proteins across 50 eukaryotes: new insights into subfamily classification and expansion. *Sci Rep-UK*. **5**, 11037 (2015).

11. Feller, A., Machemer, K., Braun, E. L. & Grotewold, E. Evolutionary and comparative analysis of MYB and bHLH plant transcription factors. *Plant J.* **66**, 94-116 (2011).

12. Prouse, M. B. & Campbell, M. M. The interaction between MYB proteins and their target DNA binding sites. *BBA-Gene Regul. Mech.* **1819**, 67-77 (2012).

13. Ogata, K. *et al*. The cavity in the hydrophobic core of MYB DNA-binding domain is reserved for DNA recognition and trans-activation. *Nat. Struct. Biol.* **3**, 178-187 (1996).

14. Wang, H., Wang, H., Shao, H. & Tang, X. Recent advances in utilizing transcription factors to improve plant abiotic stress tolerance by transgenic technology. *Front. Plant Sci.* **7, (**2016).

15. Wang, B. *et al*. Structural insights into target DNA recognition by R2R3-MYB transcription factors. *Nucleic Acids Res.* **48**, 460-471 (2020).

16. Xie, Z. *et al*. Regulation of cell proliferation in the stomatal lineage by the *Arabidopsis* MYB FOUR LIPS via direct targeting of core cell cycle genes. *Plant Cell* **22**, 2306-2321 (2010).

17. Jin, H. & Martin, C. Multifunctionality and diversity within the plant MYB-gene family. *Plant Mol. Biol.* **41**, 577-585 (1999).

18. Li, P. *et al*. MYB Superfamily in *Brassica napus*: evidence for hormone-mediated expression profiles, large expansion, and functions in root hair development. *Biomolecules* **10,** (2020e).

19. Tombuloglu, H. Genome-wide identification and expression analysis of R2R3, 3R-and 4R-MYB transcription factors during lignin biosynthesis in flax (*Linum usitatissimum*). *Genomics* **112**, 782-795 (2020).

20. Paz-Ares, J., Ghosal, D., Wienand, U., Peterson, P. A. & Saedler, H. The regulatory c1 locus of *Zea mays* encodes a protein with homology to myb proto-oncogene products and with structural similarities to transcriptional activators. *EMBO J.* **6**, 3553-3558 (1987).

21. Wilkins, O., Nahal, H., Foong, J., Provart, N. J. & Campbell, M. M. Expansion and diversification of the Populus R2R3-MYB family of transcription factors. *Plant Physiol.* **149**, 981-993 (2009).

22. Du, H. *et al*. Genome-wide analysis of the MYB transcription factor superfamily in soybean. *BMC Plant Biol.* **12**, 106 (2012b).

23. Wang, N. *et al*. A comparative genome-wide analysis of the R2R3-MYB gene family among four gossypium species and their sequence variation and association with fiber quality traits in an interspecific *G. hirsutum* x *G. barbadense* population. *Front. Genet.* **10, (**2019b).

24. Gocal, G. F. *et al*. Long-day up-regulation of a *GAMYB* gene during *Lolium temulentum* inflorescence formation. *Plant Physiol.* **119**, 1271-1278 (1999).

25. Jin, H. *et al*. Transcriptional repression by *AtMYB4* controls production of UV-protecting sunscreens in *Arabidopsis*. *EMBO J.* **19**, 6150-6161 (2000).

26. Jung, C. *et al*. Overexpression of *AtMYB44* enhances stomatal closure to confer abiotic stress tolerance in transgenic *Arabidopsis*. *Plant Physiol.* **146**, 623-635 (2008).

27. Zhang, P. *et al*. The R2R3-MYB transcription factor MYB49 regulates cadmium accumulation. *Plant Physiol.* **180**, 529-542 (2019).

28. Gong, Q. *et al*. SUMOylation of MYB30 enhances salt tolerance by elevating alternative respiration via transcriptionally upregulating AOX1a in *Arabidopsis*. *Plant J.* **102**, 1157-1171 (2020).

29. He, J. *et al*. An R2R3 MYB transcription factor confers brown planthopper resistance by regulating the phenylalanine ammonia-lyase pathway in rice. *P. Natl. Acad. Sci. USA*. **117**, 271-277 (2020).

30. Stracke, R. *et al*. Differential regulation of closely related R2R3-MYB transcription factors controls flavonol accumulation in different parts of the *Arabidopsis thaliana* seedling. *Plant J.* **50**, 660-677 (2007).

31. Tian, J. *et al*. Characteristics of dihydroflavonol 4-reductase gene promoters from different leaf colored *Malus crabapple* cultivars. *Hortic. Res.* **4**, 17070 (2017).

32. Wang, L. *et al*. A MYB/bHLH complex regulates tissue-specific anthocyanin biosynthesis in the inner pericarp of red-centered kiwifruit *Actinidia chinensis* cv. Hongyang. *Plant J.* **99**, 359-378 (2019).

33. Ding, B. *et al*. Two MYB proteins in a self-organizing activator-inhibitor system produce spotted pigmentation patterns. *Curr. Biol.* **30**, 802-814 (2020).

34. Yan, S. *et al*. Anthocyanin fruit encodes an R2R3-MYB transcription factor, *SlAN2-like*, activating the transcription of *SlMYBATV* to fine-tune anthocyanin content in tomato fruit. *New Phytol.* **225**, 2048-2063 (2020).

35. Chen, C. *et al*. MYB transcription repressors regulate plant secondary metabolism. *Crit. Rev. Plant Sci.* **38**,159-170 (2019).

36. Lee, D. K., Geisler, M. & Springer, P. S. *LATERAL ORGAN FUSION1* and *LATERAL ORGAN FUSION2* function in lateral organ separation and axillary meristem formation in *Arabidopsis*. *Development* **136**, 2423-2432 (2009).

37. Mandaokar, A. & Browse, J. MYB108 acts together with MYB24 to regulate jasmonate-mediated stamen maturation in *Arabidopsis. Plant Physiol.* **149**, 851-862 (2009).

38. Sun, W. *et al*. Cotton fiber elongation requires the transcription factor *GhMYB212* to regulate sucrose transportation into expanding fibers. *New Phytol.* **222**, 864-881 (2019).

39. Baumann, K. *et al*. Control of cell and petal morphogenesis by R2R3 MYB transcription factors. *Development* **134**, 1691-1701 (2007).

40. Tominaga, R., Iwata, M., Okada, K. & Wada, T. Functional analysis of the epidermal-specific MYB genes *CAPRICE* and *WEREWOLF* in *Arabidopsis*. *Plant Cell* **19**, 2264-2277 (2007).

41. Yan, Y. *et al*. MYB30 is a key negative regulator of *Arabidopsis* photomorphogenic development that promotes PIF4 and PIF5 protein accumulation in the light. *Plant Cell* **32**, 2196-2215 (2020).

42. Naing, A. H. & Kim, C. K. Roles of R2R3-MYB transcription factors in transcriptional regulation of anthocyanin biosynthesis in horticultural plants. *Plant Mol. Biol.* **98**, 1-18 (2018).

43. Ma, D. & Constabel, C. P. MYB repressors as regulators of phenylpropanoid metabolism in plants. *Trends Plant Sci.* **24**, 275-289 (2019).

44. Cao, Y., Li, K., Li, Y., Zhao, X. & Wang, L. MYB Transcription factors as regulators of secondary metabolism in plants. *Biology (Basel)* **9**, 61 (2020).

45. Wen, W., Alseekh, S. & Fernie, A.R. Conservation and diversification of flavonoid metabolism in the plant kingdom. *Curr. Opin. Plant Biol.* **55**, 100-108 (2020).

46. Yang, Y. & Liu, H. Coordinated shoot and root responses to light signaling in *Arabidopsis*. *Plant Commun.* **1**, 100026 (2020).

47. LaFountain, A. M. & Yuan, Y. W. Repressors of anthocyanin biosynthesis. *New Phytol.* **231**, 933-949 (2021).

48. Grotewold, E., Sainz, M. S., Tagliani, L., Hernandez, J. M., Bowen, B. & Chandler, V. L. Identification of the residues in the Myb domain of maize C1 that specify the interaction with the bHLH cofactor R. *Proc. Natl. Acad. Sci. USA*. **97**, 13579–13584 (2000).

49. Du, H., Feng, B. R., Yang, S. S., Huang, Y. B. & Tang, Y. X. The R2R3-MYB transcription factor gene family in maize. *PloS One* **7**, e37463-e37463 (2012).

50. Pu, X. *et al*. Genome-wide analysis of the MYB transcription factor superfamily in *Physcomitrella patens*. *Int. J. Mol. Sci.* **21,** (2020).

51. Liang, Z. *et al*. *Mesostigma viride* genome and transcriptome provide insights into the origin and evolution of Streptophyta. *Adv. Sci.* **7**, 1901850 (2020).

52. Li, Y. *et al*. Genome-wide analysis and expression profiles of the StR2R3-MYB transcription factor superfamily in potato (*Solanum tuberosum* L.). *Int. J. Biol. Macromol.* **148**, 817-832 (2020).

53. Pucker, B. Automatic identification and annotation of MYB gene family members in plants. *bioRxiv*.

54. Millard, P. S., Kragelund, B. B. & Burow, M. R2R3 MYB transcription factors - Functions outside the DNA-Binding domain. *Trends Plant Sci.* **24**, 934-946 (2019).

55. Sarkar, M. A. R., Watanabe, S., Suzuki, A., Hashimoto, F. & Anai, T. Identification of novel MYB transcription factors involved in the isoflavone biosynthetic pathway by using the combination screening system with agroinfiltration and hairy root transformation. *Plant Biotechnol-NAR.* **36**, 241-251 (2019).

56. Jiang, C., Gu, X. & Peterson, T. Identification of conserved gene structures and carboxy-terminal motifs in the MYB gene family of *Arabidopsis* and *Oryza sativa* L. ssp. *indica*. *Genome Biol.* **5**, R46 (2004).

57. Matus, J. T., Aquea, F. & Arce-Johnson, P. Analysis of the grape MYB R2R3 subfamily reveals expanded wine quality-related clades and conserved gene structure organization across *Vitis* and *Arabidopsis* genomes. *BMC Plant Biol.* **8**, 83-83 (2008).

58. Li, Q., Zhang, C., Li, J., Wang, L. & Ren, Z. Genome-wide identification and characterization of R2R3MYB family in *Cucumis sativus*. *PloS One* **7**, e47576-e47576 (2012).

59. Bowman, J. L. *et al*. Insights into land plant evolution garnered from the *Marchantia polymorpha* genome. *Cell* **171**, 287-304.e215 (2017).

60. Chang, X. *et al*. Origins and stepwise expansion of R2R3-MYB transcription factors for the terrestrial adaptation of plants. *Front. Plant Sci.* **11, (**2020).

61. He, C. *et al*. Mining MYB transcription factors from the genomes of orchids (*Phalaenopsis* and *Dendrobium*) and characterization of an orchid R2R3-MYB gene involved in water-soluble polysaccharide biosynthesis. *Sci*. *Rep*. **9**, 13818 (2019).

62. Dias, A. P., Braun, E. L., McMullen, M. D. & Grotewold, E. Recently duplicated maize R2R3 MYB genes provide evidence for distinct mechanisms of evolutionary divergence after duplication. *Plant Physiol.* **131**, 610-620 (2003).

63. Peng, X., Liu, H., Wang, D. & Shen, S. Genome-wide identification of the *Jatropha curcas* MYB family and functional analysis of the abiotic stress responsive gene *JcMYB2*. *BMC Genomics* **17**, 251 (2016).

64. Liu, C. *et al*. Genome-wide organization and expression profiling of the R2R3-MYB transcription factor family in pineapple (*Ananas comosus*). *BMC Genomics* **18**, 503 (2017).

65. Zhou, Q. *et al*. MYB transcription factors in alfalfa (*Medicago sativa*): genome-wide identification and expression analysis under abiotic stresses. *PeerJ* **7**, e7714 (2019).

66. Zhou, W., Zhang, Q., Sun, Y., Yang, L. & Wang, Z. Genome-wide identification and characterization of R2R3-MYB family in *Hypericum perforatum* under diverse abiotic stresses. *Int*. *J*. *Biol*. *Macromol*. **145**, 341-354 (2020).

67. Li, H. Y. *et al*. Genome-wide identification, classification, and expression profiling reveals R2R3-MYB Transcription factors related to monoterpenoid biosynthesis in *Osmanthus fragrans*. *Genes (Basel)* **11,** (2020).

68. Carretero-Paulet, L. *et al*. Genome-wide classification and evolutionary analysis of the bHLH family of transcription factors in Arabidopsis, Poplar, Rice, Moss, and Algae. *Plant Physiol.* **153**, 1398-1412 (2010).

69. Vekemans, D. *et al*. Gamma paleohexaploidy in the stem lineage of core eudicots: significance for MADS-box gene and species diversification. *Mol. Biol. Evol.* **29**, 3793-3806 (2012).

70. Cenci, A. & Rouard, M. Evolutionary analyses of GRAS transcription factors in angiosperms. *Front. Plant Sci.* **8,** (2017).

71. Ohta, M., Matsui, K., Hiratsu, K., Shinshi, H. & Ohme-Takagi, M. Repression domains of class II ERF transcriptional repressors share an essential motif for active repression. *Plant Cell.* **13*,*** 1959-1968 (2001).

72. Matsui, K., Umemura, Y. & Ohme-Takagi, M. AtMYBL2, a protein with a single MYB domain, acts as a negative regulator of anthocyanin biosynthesis in Arabidopsis. *Plant J*. **55,** 954-967 (2008).

73. Rodrigues, J. A., Espley, R. V. & Allan, A. C. Genomic analysis uncovers functional variation in the C-terminus of anthocyanin-activating MYB transcription factors. *Hortic. Res.***8,** 77(2021).

74. Chen, L., Hu, B., Qin, Y., Hu, G. & Zhao, J. Advance of the negative regulation of anthocyanin biosynthesis by MYB transcription factors. *Plant Physiol. Bioch.* **136**, 178-187 (2019).

75. Du, H. *et al*. Biochemical and molecular characterization of plant MYB transcription factor family. *Biochemistry (Mosc)* **74**, 1-11 (2009).

76. Ambawat, S. Sharma, P. Yadav, N.R. & Yadav, R.C. MYB transcription factor genes as regulators for plant responses: an overview. *Physiol. Mol. Biol. Plants.* **19**, 307-321 (2013).

77. Liu, J. Osbourn, A. & Ma, P. MYB transcription factors as regulators of phenylpropanoid metabolism in plants. *Mol. Plant*. **8**, 689-708 (2015).

78. Lee, M. M. & Schiefelbein, J. Developmentally distinct MYB genes encode functionally equivalent proteins in *Arabidopsis*. *Development*. **128**, 1539-1546 (2001).

79. Higginson, T., Li, S. F. & Parish, R. W. *AtMYB103* regulates tapetum and trichome development in *Arabidopsis thaliana*. *Plant J.* **35**,177-192 (2003).

80. Oshima, Y. *et al*. MIXTA-like transcription factors and WAX INDUCER1/SHINE1 coordinately regulate cuticle development in *Arabidopsis* and *Torenia fournieri*. *Plant Cell* **25**, 1609-1624 (2013).

81. Shin, B. *et al*. *AtMYB21*, a gene encoding a flower-specific transcription factor, is regulated by COP1. *Plant J.* **30**, 23-32 (2002).

82. Steiner-Lange, S. *et al*. Disruption of *Arabidopsis thaliana* MYB26 results in male sterility due to non-dehiscent anthers. *Plant J.* **34**, 519-528 (2003).

83. Li, J. *et al*. Ectopic expression of a R2R3 MYB transcription factor of dove tree (*Davidia involucrata*) aggravates seed abortion in *Arabidopsis thaliana*. *Funct. Plant Biol.* **47**, 454-463 (2020).

84. Li, S. F. *et al*. MYB-bHLH-TTG1 regulates *Arabidopsis* seed coat biosynthesis pathways directly and indirectly via multiple tiers of transcription factors. *Plant Cell Physiol*. **61**, 1005-1018 (2020).

85. Tan, H. *et al*. A crucial role of GA-Regulated flavonol biosynthesis in root growth of *Arabidopsis*. *Mol. Plant*. **12**, 521-537 (2019).

86. Wang, W., Ryu, K. H., Barron, C. & Schiefelbein, J. Root epidermal cell patterning is modulated by a critical residue in the WEREWOLF transcription factor. *Plant Physiol.* **181**, 1239-1256 (2019).

87. Xu, B. *et al*. The land plant-specific MIXTA-MYB lineage is implicated in the early evolution of the plant cuticle and the colonization of land. *New Phytol*. **229**, 2324-2338 (2020).

88. Perez-Rodriguez, M., Jaffe, F. W., Butelli, E., Glover, B. J. & Martin, C. 2005. Development of three different cell types is associated with the activity of a specific MYB transcription factor in the ventral petal of *Antirrhinum majus* flowers. *Development* **132**, 359-370 (2005).

89. Zhou, C. & Li, C. A novel R2R3-MYB transcription factor *BpMYB106* of Birch (*Betula platyphylla*) confers increased photosynthesis and growth rate through up-regulating photosynthetic gene expression. *Front. Plant Sci.* **7**, 315 (2016).

90. Wu, H. *et al*. Genetics and evolution of MIXTA genes regulating cotton lint fiber development. *New Phytol.* **217**, 883-895 (2018).

91. Millar, A. A. & Gubler, F. The *Arabidopsis* *GAMYB-Like* Genes, *MYB33* and *MYB65*, are microRNA-regulated genes that redundantly facilitate anther development. *Plant Cell* **17**, 705-721 (2005).

92. Shen, X., Hu, Z., Xiang, X., Xu, L. & Cao, J. Overexpression of a stamen-specific R2R3-MYB gene *BcMF28* causes aberrant stamen development in transgenic *Arabidopsis*. *Biochem. Bioph. Res. Co.* **518**, 726-731 (2019).

93. Aya, K. *et al*. Gibberellin modulates anther development in rice via the transcriptional regulation of GAMYB. *Plant Cell* **21**, 1453-1472 (2009).

94. Liang, Y. *et al*. *MYB97*, *MYB101* and *MYB120* function as male factors that control pollen tube-synergid interaction in *Arabidopsis thaliana* fertilization. *PLoS Genet.* **9**, e1003933-e1003933 (2013).

95. Rabiger, D. S. & Drews, G. N. MYB64 and MYB119 are required for cellularization and differentiation during female gametogenesis in *Arabidopsis thaliana*. *PLoS Genet.* **9**, e1003783 (2013).

96. Hisanaga, T. *et al*. A cis-acting bidirectional transcription switch controls sexual dimorphism in the liverwort. *Embo*. *J.* **38**, e100240 (2019).

97. Aya, K. et al. The Gibberellin perception system evolved to regulate a pre-existing GAMYB-mediated system during land plant evolution. *Nat. Commun.* **2**(2011).

98. Huang, C. *et al*. *NbPHAN*, a MYB transcriptional factor, regulates leaf development and affects drought tolerance in *Nicotiana benthamiana*. *Physiol. Plant.* **149**, 297-309 (2013).

99. Ge, L. & Chen, R. PHANTASTICA regulates leaf polarity and petiole identity in *Medicago truncatula*. *Plant Signal. Behav.* **9**, e28121-e28121 (2014).

100. Tsiantis, M., Schneeberger, R., Golz, J. F., Freeling, M. & Langdale, J. A. The maize rough sheath2 gene and leaf development programs in monocot and dicot plants. *Science* **284**, 154-156 (1999).

101. Morimoto, R., Nishioka, E., Murai, K. & Takumi, S. Functional conservation of wheat orthologs of maize *rough sheath1* and *rough sheath2* genes. *Plant Mol. Biol.* **69**, 273-285 (2009).

102. Zoulias, N., Koenig, D., Hamidi, A., McCormick, S. & Kim, M. A role for PHANTASTICA in medio-lateral regulation of adaxial domain development in tomato and tobacco leaves. *Ann. Bot-London.* **109**, 407-418 (2011).

103. Hong, S. H. *et al*. CRY1 inhibits COP1-mediated degradation of BIT1, a MYB transcription factor, to activate blue light-dependent gene expression in *Arabidopsis*. *Plant J.* **55**, 361-371 (2008).

104. Fernández-Marcos, M. *et al*. Control of *Arabidopsis* lateral root primordium boundaries by MYB36. *New Phytol.* **213**, 105-112 (2017).

105. Yasui, Y. *et al*. GEMMA CUP-ASSOCIATED MYB1, an ortholog of axillary meristem regulators, is essential in vegetative reproduction in *Marchantia polymorpha*. *Curr. Biol.* **29**, 3987-3995 (2019).

106. Leech, M. J., Kammerer, W., Cove, D. J., Martin, C. & Wang, T. L. Expression of myb-related genes in the moss, *Physcomitrella patens*. *Plant J.* 3, 51-61(1993).

107. Mandaokar, A. *et al*. Transcriptional regulators of stamen development in Arabidopsis identified by transcriptional profiling. *Plant J.* **46**, 984-1008 (2006).

108. Cheng, H. *et al*. Gibberellin acts through jasmonate to control the expression of MYB21, MYB24, and MYB57 to promote stamen filament growth in *Arabidopsis*. *PLoS Genet.* **5**, e1000440 (2009).

109. Shan, X. *et al*. The spatio-temporal biosynthesis of floral flavonols is controlled by differential phylogenetic MYB regulators in *Freesia hybrida*. *New Phytol*. **228**, 1864-1879 (2020).

110. Yang, Z. *et al*. MYB21 interacts with MYC2 to control the expression of terpene synthase genes in flowers of *Freesia hybrida* and *Arabidopsis thaliana*. *J. Exp. Bot.* **71**, 4140-4158 (2020).

111. Nakatsuka, T. *et al*. Identification and characterization of R2R3-MYB and bHLH transcription factors regulating anthocyanin biosynthesis in Gentian flowers. *Plant Cell Physiol.* **49**, 1818-1829 (2008).

112. Li, J., An, Y. & Wang, L. Transcriptomic analysis of *Ficus carica* peels with a focus on the key genes for anthocyanin biosynthesis. *Int. J. Mol. Sci.* **21,** (2020).

113. Wang, Y. *et al*. The R2R3-MYB transcription factor *MdMYB24-like* is involved in methyl jasmonate-induced anthocyanin biosynthesis in apple. *Plant Physiol. Bioch.* **139**, 273-282 (2019).

114. Spitzer-Rimon, B. *et al*. *EOBII*, a gene encoding a flower-specific regulator of phenylpropanoid volatiles' biosynthesis in petunia. *Plant Cell* **22**, 1961-1976 (2010).

115. Yang, S. W., Jang, I. C., Henriques, R. & Chua, N. H. FAR-RED ELONGATED HYPOCOTYL1 and FHY1-LIKE associate with the *Arabidopsis* transcription factors LAF1 and HFR1 to transmit phytochrome A signals for inhibition of hypocotyl elongation. *Plant Cell* **21**, 1341-1359 (2009).

116. Gibbs, D. J. *et al*. *AtMYB93* is a novel negative regulator of lateral root development in *Arabidopsis*. *New Phytol.* **203**, 1194-1207 (2014).

117. Wang, Z. *et al*. Transcriptional regulation of MdPIN3 and MdPIN10 by MdFLP during apple self-rooted stock adventitious root gravitropism. *BMC Plant Biol.* **19, (**2019).

118. Yuan, Y. *et al*. R2R3 MYB-dependent auxin signalling regulates trichome formation, and increased trichome density confers spider mite tolerance on tomato. *Plant Biotechnol. J.* **19**, 138-152 (2020)*.*

119. Zhu, J. *et al*. Defective in tapetal development and function 1 is essential for anther development and tapetal function for microspore maturation in *Arabidopsis*. *Plant J.* **55**, 266-277 (2008).

120. Zhang, Y., Cao, G., Qu, L. J. & Gu, H. Characterization of *Arabidopsis* MYB transcription factor gene *AtMYB17* and its possible regulation by LEAFY and AGL15. *J. Genet. Genomics* **36**, 99-107 (2009).

121. Liang, G., He, H., Li, Y., Ai, Q. & Yu, D. MYB82 functions in regulation of trichome development in Arabidopsis. *J. Exp. Bot.* **65**, 3215-3223 (2014).

122. Zhang, Y. *et al*. *PaMYB82* from *Platanus acerifolia* regulates trichome development in transgenic *Arabidopsis*. *Plant Sci.* **287, (**2019).

123. Yang, A., Dai, X. & Zhang, W. H. A R2R3-type MYB gene, *OsMYB2*, is involved in salt, cold, and dehydration tolerance in rice. *J. Exp. Bot.* **63**, 2541-2556 (2012).

124. Zhao, Y. *et al*. The wheat MYB Transcription factor *TaMYB(31)* is involved in drought stress responses in *Arabidopsis*. *Front. Plant Sci.* **9**, 1426-1426 (2018).

125. Agarwal, P., Mitra, M., Banerjee, S. & Roy, S. MYB4 transcription factor, a member of R2R3-subfamily of MYB domain protein, regulates cadmium tolerance via enhanced protection against oxidative damage and increases expression of *PCS1* and *MT1C* in *Arabidopsis*. *Plant Sci.* **297, (**2020).

126. Fichman, Y. *et al*. MYB30 orchestrates systemic reactive oxygen signaling and plant acclimation. *Plant Physiol.* **184**, pp.00859.2020 (2020).

127. Yao, L. *et al*. The R2R3-MYB transcription factor *BnaMYB111L* from rapeseed modulates reactive oxygen species accumulation and hypersensitive-like cell death. *Plant Physiol. Bioch.* **147**, 280-288 (2020).

128. Li, C., Ng, C. K. Y. & Fan, L. M. MYB transcription factors, active players in abiotic stress signaling. *Environ. Exp. Bot.* **114**, 80-91 (2015).

129. Ng, D. W. K., Abeysinghe, J. K. & Kamali, M. Regulating the regulators: the control of transcription factors in plant defense signaling. *Int. J. Mol. Sci.* **19,** (2018.).

130. Cominelli, E. *et al*. 2005. A guard-cell-specific MYB transcription factor regulates stomatal movements and plant drought tolerance. *Curr. Biol.* **15**, 1196-1200 (2005).

131. Seo, P. J. *et al*. The MYB96 transcription factor mediates abscisic acid signaling during drought stress response in *Arabidopsis*. *Plant Physiol.* **151**, 275-289 (2009).

132. Seo, P. J. *et al*. The MYB96 transcription factor regulates cuticular wax biosynthesis under drought conditions in *Arabidopsis*. *Plant Cell* **23**, 1138-1152 (2011).

133. Marino, D. *et al*. *Arabidopsis* ubiquitin ligase MIEL1 mediates degradation of the transcription factor MYB30 weakening plant defence. *Nat. Commun.* **4**, 1476 (2013).

134. Rusconi, F. *et al*. The *Arabidopsis thaliana* MYB60 promoter provides a tool for the spatio-temporal control of gene expression in stomatal guard cells. *J. Exp. Bot.* **64**, 3361-3371 (2013).

135. Lee, H. G. & Seo, P. J. The MYB96–HHP module integrates cold and abscisic acid signaling to activate the CBF–COR pathway in *Arabidopsis*. *Plant J.* **82**, 962-977 (2015).

136. Liao, C. Zheng, Y. & Guo, Y. MYB30 transcription factor regulates oxidative and heat stress responses through ANNEXIN-mediated cytosolic calcium signaling in *Arabidopsis*. *New Phytol.* **216**, 163-177 (2017).

137. Yan, J. *et al*. Comparative transcriptome analysis of celery leaf blades identified an R2R3-MYB transcription factor that regulates apigenin metabolism. *J. Agr. Food Chem.* **67**, 5265-5277 (2019).

138. Kirik, V., Kölle, K., Miséra, S. & Bäumlein, H. Two novel MYB homologues with changed expression in late embryogenesis-defective *Arabidopsis* mutants. *Plant Mol. Biol.* **37**, 819-827 (1998).

139. Ding, Z. *et al*. Transgenic expression of *MYB15* confers enhanced sensitivity to abscisic acid and improved drought tolerance in *Arabidopsis thaliana*. *J. Genet. Genomics* **36**, 17-29 (2009).

140. Chen, Y. *et al*. *AtMYB14* regulates cold tolerance in *Arabidopsis*. *Plant Mol. Biol. Report*. **31**, 87-97 (2013).

141. Chezem, W. R., Memon, A., Li, F. S., Weng, J. K. & Clay, N. K. SG2-Type R2R3-MYB transcription factor MYB15 controls defense-induced lignification and basal immunity in *Arabidopsis*. *Plant Cell* **29**, 1907-1926 (2017).

142. Hoang, M. H. T. *et al*. Phosphorylation by *AtMPK6* is required for the biological function of *AtMYB41* in Arabidopsis. *Biochem. Bioph. Res. Co.* **422**, 181-186 (2012).

143. Xu, R. *et al*. Salt-induced transcription factor MYB74 is regulated by the RNA-directed DNA methylation pathway in *Arabidopsis*. *J. Exp. Bot.* **66**, 5997-6008 (2015).

144. Devaiah, B. N., Madhuvanthi, R., Karthikeyan, A. S. & Raghothama, K. G. Phosphate starvation responses and gibberellic acid biosynthesis are regulated by the MYB62 transcription factor in *Arabidopsis*. *Mol. Plant*. **2**, 43-58 (2009).

145. Baek, D. *et al*. Regulation of miR399f transcription by *AtMYB2* affects phosphate starvation responses in *Arabidopsis*. *Plant Physiol.* **161**, 362-373 (2013).

146. Chou, M. L. *et al*. The Direct involvement of dark-induced Tic55 protein in chlorophyll catabolism and its indirect role in the MYB108-NAC signaling pathway during leaf senescence in *Arabidopsis thaliana*. *Int. J. Mol. Sci.* **19**, 1854 (2018).

147. Zhao, Y. *et al*. The ABA receptor PYL8 promotes lateral root growth by enhancing MYB77-dependent transcription of auxin-responsive genes. *Sci. Signal.* **7**, ra53-ra53 (2014).

148. Nguyen, N. H. & Cheong, J. J. The *AtMYB44* promoter is accessible to signals that induce different chromatin modifications for gene transcription. *Plant Physiol. Bioch.* **130**, 14-19 (2018).

149. Nishida, S., Kakei, Y., Shimada, Y. & Fujiwara, T. Genome-wide analysis of specific alterations in transcript structure and accumulation caused by nutrient deficiencies in *Arabidopsis thaliana*. *Plant J.* **91**, 741-753 (2017).

150. Palmer, C. M., Hindt, M. N., Schmidt, H., Clemens, S. & Guerinot, M. L. MYB10 and MYB72 are required for growth under iron-limiting conditions. *PLoS Genet.* **9**, e1003953 (2013).

151. Cui, M. H. *et al*. An *Arabidopsis* R2R3-MYB transcription factor, *AtMYB20*, negatively regulates type 2C serine/threonine protein phosphatases to enhance salt tolerance. *FEBS Lett.* **587**, 1773-1778 (2013).

152. Wang, T. *et al*. Salt-related MYB1 coordinates abscisic acid biosynthesis and signaling during salt stress in *Arabidopsis*. *Plant Physiol.* **169**, 1027-1041 (2015).

153. Agarwal, M. *et al*. A R2R3 type MYB transcription factor is involved in the cold regulation of CBF genes and in acquired freezing tolerance. *J. Biol. Chem.* **281**, 37636-37645 (2006).

154. He, Y. *et al*. Ectopic expression of a wheat MYB transcription factor gene, *TaMYB73*, improves salinity stress tolerance in *Arabidopsis thaliana*. *J. Exp. Bot.* **63**, 1511-1522 (2012).

155. Chen, S. *et al*. *NtMYB4* and *NtCHS1* are critical factors in the regulation of flavonoid biosynthesis and are involved in salinity responsiveness. *Front. Plant Sci.* **10, (**2019).

156. Liao, W., Yang, Y., Li, Y., Wang, G. & Peng, M. Genome-wide identification of cassava R2R3 MYB family genes related to abscission zone separation after environmental-stress-induced abscission. *Sci. Rep.* **6**, 32006-32006 (2016).

157. Fang, Q. *et al*. A salt-stress-regulator from the Poplar R2R3 MYB family integrates the regulation of lateral root emergence and ABA signaling to mediate salt stress tolerance in *Arabidopsis*. *Plant Physiol. Bioch.* **114**, 100-110 (2017).

158. Zhang, X., Chen, L., Shi, Q. & Ren, Z. *SIMYB102*, an R2R3-type MYB gene, confers salt tolerance in transgenic tomato. *Plant Sci.* **291, (**2020).

159. Zhang, Z. *et al*. An R2R3 MYB transcription factor in wheat, *TaPIMP1*, mediates host resistance to *Bipolaris sorokiniana* and drought stresses through regulation of defense- and stress-related genes. *New Phytol.* **196**, 1155-1170 (2012).

160. Zhang, Z. *et al*. Opposing control by transcription factors MYB61 and MYB3 increases freezing tolerance by relieving C-Repeat binding factor suppression. *Plant Physiol.* **172**, 1306-1323 (2016).

161. Zhang, S. *et al*. *RhMYB108*, an R2R3-MYB transcription factor, is involved in ethylene- and JA-induced petal senescence in rose plants. *Hortic. Res.* **6**, 131 (2019).

162. Shukla, P. S., Agarwal, P., Gupta, K. & Agarwal, P. K. Molecular characterization of an MYB transcription factor from a succulent halophyte involved in stress tolerance. *Aob Plants* **7**, plv054 (2015).

163. Gao, F. *et al*. Identification, isolation and expression analysis of eight stress-related R2R3-MYB genes in tartary buckwheat (*Fagopyrum tataricum*). *Plant Cell Rep*. **35**, 1385-1396 (2016).

164. Hu, D. G. *et al*. The R2R3-MYB transcription factor *MdMYB73* is involved in malate accumulation and vacuolar acidification in apple. *Plant J.* **91**, 443-454 (2017).

165. Zhao, Y. *et al*. Over-expression of an R2R3 MYB Gene, *GhMYB73*, increases tolerance to salt stress in transgenic *Arabidopsis*. *Plant Sci.* **286**, 28-36 (2019).

166. Lee, T. G. *et al*. A MYB transcription factor (*TaMyb1*) from wheat roots is expressed during hypoxia: roles in response to the oxygen concentration in root environment and abiotic stresses. *Physiol. Plant.* **129**, 375-385 (2007).

167. Garg, B., Lata, C. & Prasad, M. A study of the role of gene TaMYB2 and an associated SNP in dehydration tolerance in common wheat. *Mol. Biol. Rep.* **39,** 10865-10871 (2012).

168. Cao, Z. H., Zhang, S. Z., Wang, R. K., Zhang, R. F. & Hao, Y. J. Genome wide analysis of the apple myb transcription factor family allows the identification of *MdoMYB121* gene confering abiotic stress tolerance in plants. *PLoS One* **8**, e69955 (2013).

169. Wang, R. K., Cao, Z. H. & Hao, Y. J. Overexpression of a R2R3 MYB gene *MdSIMYB1* increases tolerance to multiple stresses in transgenic tobacco and apples. *Physiol. Plantarum*. **150**, 76-87 (2014).

170. Zhang, L. *et al*. Characterization of a wheat R2R3-MYB transcription factor gene, *TaMYB19*, involved in enhanced abiotic stresses in *Arabidopsis*. *Plant Cell Physiol.* **55**, 1802-1812 (2014).

171. Dong, W. *et al*. The *Medicago truncatula* R2R3-MYB transcription factor gene *MtMYBS1* enhances salinity tolerance when constitutively expressed in *Arabidopsis thaliana*. *Biochem. Bioph. Res. Co.* **490**, 225-230 (2017).

172. Wei, H. *et al*. Chicory R2R3-MYB transcription factors *CiMYB5* and *CiMYB3* regulate fructan 1-exohydrolase expression in response to abiotic stress and hormonal cues. *J. Exp. Bot.* **68**, 4323-4338 (2017).

173. Geng, D. *et al*. *MdMYB88* and *MdMYB124* enhance drought tolerance by modulating root vessels and cell walls in apple. *Plant Physiol.* **178**, 1296-1309 (2018).

174. He, Y. *et al*. Overexpression of a novel transcriptional repressor *GmMYB3a* negatively regulates salt-alkali tolerance and stress-related genes in soybean. *Biochem. Bioph. Res. Co.* **498**, 586-591 (2018).

175. Xie, Y. *et al*. An atypical R2R3 MYB transcription factor increases cold hardiness by CBF-dependent and CBF-independent pathways in apple. *New Phytol.* **218**, 201-218 (2018).

176. Meng, C. & Sui, N. Overexpression of maize MYB-IF35 increases chilling tolerance in Arabidopsis. *Plant Physiol. Bioch.* **135**, 167-173 (2019).

177. Xing, C., Liu, Y., Zhao, L., Zhang, S. & Huang, X. A novel MYB transcription factor regulates ascorbic acid synthesis and affects cold tolerance. *Plant Cell Environ.* **42**, 832-845 (2019).

178. He, Y. *et al*. Functional activation of a novel R2R3-MYB protein gene, *GmMYB68*, confers salt-alkali resistance in soybean (*Glycine max* L.). *Genome* **63**, 13-26 (2020b).

179. An, C. *et al*. Overexpression of *CmMYB15* provides chrysanthemum resistance to aphids by regulating the biosynthesis of lignin. *Hortic. Res.* **6,** (2019).

180. Jahan, M. A. *et al*. Glyceollin transcription factor *GmMYB29A2* regulates soybean resistance to *Phytophthora sojae*. *Plant Physiol.* **183**, 530-546 (2020).

181. Yu, Y. *et al*. The grapevine R2R3-type MYB transcription factor *VdMYB1* positively regulates defense responses by activating the stilbene synthase gene 2 (VdSTS2). *BMC Plant Biol.* **19**, 478 (2019).

182. Shan, T. *et al*. The wheat R2R3-MYB transcription factor *TaRIM1* participates in resistance response against the pathogen *Rhizoctonia cerealis* infection through regulating defense genes. *Sci. Rep.* **6**, 28777 (2016).

183. Raffaele, S. *et al*. A MYB transcription factor regulates very-long-chain fatty acid biosynthesis for activation of the hypersensitive cell death response in *Arabidopsis*. *Plant Cell* **20**, 752-767 (2008).

184. Seo, P. J. & Park, C. M. MYB96-mediated abscisic acid signals induce pathogen resistance response by promoting salicylic acid biosynthesis in *Arabidopsis*. *New Phytol.* **186**, 471-483 (2010).

185. Zhu, L., Guo, J., Ma, Z., Wang, J. & Zhou, C. *Arabidopsis* transcription factor MYB102 increases plant susceptibility to aphids by substantial activation of ethylene biosynthesis. *Biomolecules* **8**, 39 (2018).

186. Segarra, G., Van der Ent, S., Trillas, I. & Pieterse, C. M. MYB72, a node of convergence in induced systemic resistance triggered by a fungal and a bacterial beneficial microbe. *Plant Biology* **11**,90-96 (2009).

187. Stringlis, I. A. *et al*. MYB72-dependent coumarin exudation shapes root microbiome assembly to promote plant health. *P. Natl. Acad. Sci. USA*. **115**, E5213-E5222 (2018).

188. Lee, M. W., Qi, M. & Yang, Y. A novel jasmonic acid-inducible rice MYB gene associates with fungal infection and host cell death. *Mol. Plant Microbe In.* **14**, 527-535 (2001).

189. Deng, Y. & Lu, S. Biosynthesis and regulation of phenylpropanoids in plants. *Front. Plant Sci.* **36,** 257-290 (2017).

190. Dong, N. Q. & Lin, H. X. Contribution of phenylpropanoid metabolism to plant development and plant-environment interactions. *J. Integr. Plant Biol.* **63,** 180-209 (2021).191. Mitreiter, S. & Gigolashvili, T. Regulation of glucosinolate biosynthesis. *J. Exp. Bot.* **72,** 70-91 (2021).

192. Wen, W., Alseekh, S. & Fernie, A. R. Conservation and diversification of flavonoid metabolism in the plant kingdom. *Curr. Opin. Plant Biol.* **55**, 100-108 (2020).

193. Liu, Y. *et al*. *StMYB44* negatively regulates anthocyanin biosynthesis at high temperatures in tuber flesh of potato. *J. Exp. Bot.* **70**, 3809-3824 (2019).

194. Zhao, Y. et al. Whole-genome resequencing-based QTL-seq identified *AhTc1* gene encoding a R2R3-MYB transcription factor controlling peanut purple testa colour. *Plant Biotechnol. J.* **18**, 96-105 (2020).

195. Xi, W., Feng, J., Liu, Y., Zhang, S. & Zhao, G. The R2R3-MYB transcription factor PaMYB10 is involved in anthocyanin biosynthesis in apricots and determines red blushed skin. *BMC Plant Biol.* **19, (**2019).

196. Lai, B. *et al*. Characterization of a novel litchi R2R3-MYB transcription factor that involves in anthocyanin biosynthesis and tissue acidification. *BMC Plant Biol.* **19**, 62 (2019).

197. Kubo, H, *et al.* Biosynthesis of riccionidins and marchantins is regulated by R2R3-MYB transcription factors in *Marchantia polymorpha*. *J. Plant Res.* 131, 849-864 (2018).

198. Cao, Y. *et al*. *PpMYB15* and *PpMYBF1* transcription factors are involved in regulating flavonol biosynthesis in peach fruit. *J. Agr. Food Chem.* **67**, 644-652 (2019).

199. Zhang, Y. *et al*. Citrus PH4-Noemi regulatory complex is involved in proanthocyanidin biosynthesis via a positive feedback loop. *J. Exp. Bot.* **71**, 1306-1321 (2020).

200. Wu, M. *et al*. *SlMYB72* regulates the metabolism of chlorophylls, carotenoids, and flavonoids in tomato fruit. *Plant Physiol.* **183**, 854-868 (2020).

201. Sun, B. *et al*. Jasmonate-inducible R2R3-MYB transcription factor regulates capsaicinoid biosynthesis and stamen development in *Capsicum*. *J. Agr. Food Chem.* **67**, 10891-10903 (2019).

202. Todesco *et al.* Genetic basis and dual adaptive role of floral pigmentation in sunflowers. bioRxiv (2021).

203. Deng, C. *et al*. 2020. *SmMYB2* promotes salvianolic acid biosynthesis in the medicinal herb *Salvia miltiorrhiza*. *J. Integr. Plant Biol*. **62**, 1688-1702 (2020).

204 Li, Y. *et al*. The R2R3-MYB factor *FhMYB5* from *Freesia hybrida* contributes to the regulation of anthocyanin and proanthocyanidin in biosynthesis. *Front. Plant Sci.* **9, (**2019).

205. Li, Y. *et al*. The conserved and particular roles of R2R3-MYB regulator *FhPAP1* from *Freesia hybrida* in flower anthocyanin biosynthesis. *Plant Cell Physiol.* **61**, 1365-1380 (2020).

206. Li, B. J. *et al*. New insight into the molecular mechanism of colour differentiation among floral segments in orchids. *Comms. Bio.* **3**, 89 (2020).

207. Anwar, M. *et al*. *NtMYB3*, an R2R3-MYB from *Narcissus*, regulates flavonoid biosynthesis. *Int. J. Mol. Sci.* **20,** (2019).208. Zhao, Q. & Dixon, R. A. Transcriptional networks for lignin biosynthesis: more complex than we thought? *Trends Plant Sci.* **16**, 227-233 (2011).

209. Sun, Y., Ren, S., Ye, S., Tian, Q. & Luo, K. Identification and functional characterization of *PtoMYB055* involved in the regulation of the lignin biosynthesis pathway in *Populus tomentosa*. *Int. J. Mol. Sci.* **21,** (2020).

210. Jiao, B., Zhao, X., Lu, W., Guo, L. & Luo, K. The R2R3 MYB transcription factor MYB189 negatively regulates secondary cell wall biosynthesis in Populus. *Tree Physiol.* **39**, 1187-1200 (2019).

211. Fornalé, S. *et al*. Down-regulation of the maize and *Arabidopsis thaliana* caffeic acid O-methyl-transferase genes by two new maize R2R3-MYB transcription factors. *Plant Mol. Biol.* **62**, 809-823 (2006).

212. Huang, J. *et al*. Genome-wide identification of R2R3-MYB transcription factors regulating secondary cell wall thickening in cotton fiber development. *Plant Cell Physiol.* **60**, 687-701 (2019).

213. Ye, Y. *et al*. CEF1/OsMYB103L is involved in GA-mediated regulation of secondary wall biosynthesis in rice. *Plant Mol. Biol.* **89**, 385-401 (2015).

214. Xue, C. *et al*. *PbrMYB169* positively regulates lignification of stone cells in pear fruit. *J. Exp. Bot.* **70**, 1801-1814 (2019).

215. Zhang, J. *et al*. EjODO1, a MYB transcription factor, regulating lignin biosynthesis in developing loquat (*Eriobotrya japonica*) fruit. *Front. Plant Sci.* **7**, 1360-1360 (2016a).

216. Seo, M. S. & Kim, J. S. Understanding of MYB transcription factors involved in glucosinolate biosynthesis in Brassicaceae. *Molecules* **22,** (2017).

217. Augustine, R., Mukhopadhyay, A. & Bisht, N. C. Targeted silencing of *BjMYB28* transcription factor gene directs development of low glucosinolate lines in oilseed *Brassica juncea*. *Plant Biotechnol. J.* **11**, 855-866 (2013).

218. Araki, R. *et al*. Novel bioresources for studies of *Brassica oleracea*: identification of a kale MYB transcription factor responsible for glucosinolate production. *Plant Biotechnol. J.* **11**, 1017-1027 (2013).

219. Liu, Y. *et al*. Fine mapping and candidate gene analysis of a seed glucosinolate content QTL, qGSL-C2, in rapeseed (*Brassica napus* L.). *Theor. Appl. Genet.* **133**, 479-490 (2020).

220. Yin, J. *et al*. Functional identification of *BpMYB21* and *BpMYB61* transcription factors responding to MeJA and SA in birch triterpenoid synthesis. *BMC Plant Biol.* **20**, 374-374 (2020).

221. Hao, X. *et al*. Tanshinone and salvianolic acid biosynthesis are regulated by *SmMYB98* in *Salvia miltiorrhiza* hairy roots. *J. Adv. Res.* **23**: 1-12 (2020).

222. Liu, L., Yang, D., Xing, B., Zhang, C. & Liang, Z. *SmMYB98b* positive regulation to tanshinones in *Salvia miltiorrhiza* Bunge hairy roots. *Plant Cell Tiss. Org.* **140**, 459-467 (2020).

223. Liu, T. *et al*. *PgMYB2*, a MeJA-responsive transcription factor, positively regulates the dammarenediol synthase gene expression in *Panax Ginseng*. *Int. J. Mol. Sci.* **20,** (2019).

224. Reddy V. A., *et al.* Spearmint R2R3−MYB transcription factor MsMYB negatively regulates monoterpene production and suppresses the expression of geranyl diphosphate synthase large subunit (MsGPPS. LSU). Plant Biotechnol*.* J*.* **15,** 1105–1119 (2017).

225. Soler, M. *et al*. The *Eucalyptus grandis* R2R3-MYB transcription factor family: evidence for woody growth-related evolution and function. *New Phytol .***206**, 1364-1377 (2015).

226. Li, X. *et al*. Genome-wide identification, evolution and functional divergence of MYB transcription factors in Chinese white pear (*Pyrus bretschneideri*). *Plant Cell Physiol.* **57**, 824-847 (2016).

227. Zhao, K. & Bartley, L. E. Comparative genomic analysis of the R2R3 MYB secondary cell wall regulators of *Arabidopsis*, poplar, rice, maize, and switchgrass. *BMC Plant Biol.* **14**,135 (2104).

228. Stracke, R., Holtgrawe, D., Schneider, J., Pucker, B., Sorensen, T. R. & Weisshaar, B. Genome-wide identification and characterisation of R2R3-MYB genes in sugar beet (*Beta vulgaris*). *BMC Plant Biol.***14,** 17 (2014).

229. Hatlestad, G. J. *et al.* The beet Y locus encodes an anthocyanin MYB-like protein that activates the betalain red pigment pathway. *Nat. Genet.* **47,** 92 (2015).

**Figure legends**

**Fig. 1** **Phylogenetic relationships of 75 plant species in which the R2R3-MYB gene family has been comprehensively analyzed.** Phylogenetic relationships among these species were obtained from the Taxonomy database (https://www.ncbi.nlm.nih.gov/taxonomy). In total, 10270 sequences of 75 plant species from Rhodophyta to angiosperms have been reported. Numbers in round brackets indicate the number of R2R3-MYB family in each species. Detailed information, including corresponding reference for each species, is provided in Supplemental Table 1. The end of statistic date is November, 2021.

**Fig. 2** **Domain structure and status of the functional characterization of R2R3-MYB transcription factors.** **a.** The R2R3 MYB transcription factor is composed of a MYB-region and non-MYB-region. The DNA-binding domain, also called the MYB-region, contains conserved R2 and R3 repeats. Normally, there is a conserved bHLH-interacting motif ([D/E] Lx2[R/K]x3Lx6Lx3R) within the first two helixes of R3 domain for interactions with bHLH proteins^48^ in most activators and some repressors^43^ to form the MYB-bHLH-WDR transcriptional complex. The protein sequences in the C-terminal region often show divergence, with one or a few typical repressor motif(s), such as the EAR motif (ERF-associated amphiphilic repression), SID motif (Sensitive to ABA and Drought 2 protein interact motif), and TLLLFR. H, Helix; T, turn; W, tryptophan; X, amino acid. * indicates the motif is not included in all R2R3-MYBs, but in some of them. **b.** Number of species for which the functions of one or more R2R3-MYB genes have been identified up to 2020. In the last decade (2011–2020), there was explosive growth in the breadth of taxa. It is noteworthy that most of the species increase is ascribed to the vast characterized orthologous MYB genes in horticultural plants with similar function, especially in phenylpropanoid biosynthesis. **c.** Number of *Arabidopsis* R2R3-MYBs for which biological role(s) has been identified. As for *Arabidopsis*, all increase belongs to the newly functioned paralogs. When a gene had different functions published in different years, we sorted it into the first published year. The dark grey bar represents the number of R2R3-MYBs with unknown (i.e., not experimentally verified) functions.

**Fig. 3 Major metabolite biosynthetic pathways regulated by *R2R3-MYBs* and simplified schemes are shown. a.** The most intensively investigated regulatory metabolites are phenylpropanoid biosynthesis-derived compounds, including flavonoids and lignins. Typical groups of flavonoids are shown in the yellow box, several representative groups for specific branches of the flavonoid biosynthesis are shown in deep yellow box, and typical groups for lignins are shown in the light blue box. More information regarding the phenylpropanoid pathway has been reviewed recently^190^. **b.** *R2R3-MYB* regulation of glucosinolate biosynthesis in S12 has primarily been studied in Brassicaceae. The synthesis pathway involves three phases, with two types of starting amino acids, which are regulated by different R2R3-MYBs. A fine review has been recently published with more details by Mitreiter and Gigolashvili^191^. **c.** The best-studied terpenoids regulated by *R2R3-MYBs* are floral volatiles.

**Fig. 4** **Phylogenetic relationships among functionally characterized plant R2R3-MYBs.** **a**. The number and percentage of *R2R3-MYBs* displaying each intron pattern as shown in Supplemental Fig. 1 in *Physcomitrella patens* (Pp), *Selaginella moellendorfii* (Sm), *Zea mays* (Zm), *Oryza sativa* (Os), *Aquilegia coerulea* (Ac), *Solanum lycopersicum* (Sl), *Solanum tuberosum* (St), *Vitis vinifera* (Vv), *Arabidopsis thaliana* (At), *Citrus sinensis* (Csi), *Medicago truncatula* (Mt), *Populus trichocarpa* (Pt), and *Brassica napus* (Bna). **b**. The maximum likelihood (ML) tree was constructed with 100 replications using JJT+G model, based on 598 nonredundant R2R3-MYBs, including 126 *A. thaliana* R2R3-MYBs^9^ and 435 functionally characterized R2R3-MYBs in other plant taxa (Supplemental Table 2) as well as representatives of 73 subfamilies in our previous results^10^. The tree was rooted using S29 (the CDC5-like protein) as the outgroup. The scale bar represents 2 substitutions per site. The detail information of the ML tree is showed in Supplemental Fig. 2. Detailed information of the sequences used for phylogenetic analyses is provided in Supplemental Table 3. The subfamily classification and nomenclature of the 598 proteins were based on that of *Arabidopsis*^9^ and our previous study^10^. The new subfamilies, with the exception of the 25 subfamilies in *Arabidopsis*^9^, are underlined, and their detail function and classification information are provided in Supplemental Table 2 and Fig. 2. The colored squares indicate the intron pattern to which each subfamily member belongs (Supplemental Fig. 1). Nodes with bootstrap values ≥70% and ≥50% are shown as black and gray dots in the phylogenetic tree, respectively.
